# Strain-stabilized superconductivity

**DOI:** 10.1038/s41467-020-20252-7

**Published:** 2021-01-04

**Authors:** J. P. Ruf, H. Paik, N. J. Schreiber, H. P. Nair, L. Miao, J. K. Kawasaki, J. N. Nelson, B. D. Faeth, Y. Lee, B. H. Goodge, B. Pamuk, C. J. Fennie, L. F. Kourkoutis, D. G. Schlom, K. M. Shen

**Affiliations:** 1 Department of Physics, Laboratory of Atomic and Solid State Physics, Cornell University, Ithaca, NY 14853 USA; 2 Platform for the Accelerated Realization, Analysis, and Discovery of Interface Materials, Cornell University, Ithaca, NY 14853 USA; 3 Department of Materials Science and Engineering, Cornell University, Ithaca, NY 14853 USA; 4 Department of Materials Science and Engineering, University of Wisconsin, Madison, WI 53706 USA; 5 School of Applied and Engineering Physics, Cornell University, Ithaca, NY 14853 USA; 6 Kavli Institute at Cornell for Nanoscale Science, Ithaca, NY 14853 USA; 7 Leibniz-Institut für Kristallzüchtung, Max-Born-Str. 2, Berlin, 12489 Germany

**Keywords:** Surfaces, interfaces and thin films, Electronic properties and materials, Superconducting properties and materials

## Abstract

Superconductivity is among the most fascinating and well-studied quantum states of matter. Despite over 100 years of research, a detailed understanding of how features of the normal-state electronic structure determine superconducting properties has remained elusive. For instance, the ability to deterministically enhance the superconducting transition temperature by design, rather than by serendipity, has been a long sought-after goal in condensed matter physics and materials science, but achieving this objective may require new tools, techniques and approaches. Here, we report the transmutation of a normal metal into a superconductor through the application of epitaxial strain. We demonstrate that synthesizing RuO_2_ thin films on (110)-oriented TiO_2_ substrates enhances the density of states near the Fermi level, which stabilizes superconductivity under strain, and suggests that a promising strategy to create new transition-metal superconductors is to apply judiciously chosen anisotropic strains that redistribute carriers within the low-energy manifold of *d* orbitals.

## Abstract (web summary)

Epitaxial strain is a promising control knob to modulate *Tc* to enhance superconductivity. Here, the authors show that a metallic oxide RuO_2_ can be turned superconducting through application of epitaxial strain in thin films grown on a (110)-oriented TiO_2_ substrate.

## Introduction

In typical weak-coupling theories of superconductivity, the effective attraction *V* between electrons is mediated by the exchange of bosons having a characteristic energy scale *ω*_B_, and superconductivity condenses below a transition temperature *T*_*c*_ parameterized as^1^:

1 $${T}_{c} \sim {\omega }_{{\rm{B}}}\exp \left(-\frac{1}{N({E}_{F})V}\right)={\omega }_{{\rm{B}}}\exp \left(-\frac{1+\lambda }{\lambda -{\mu }^{* }}\right),$$

where *N*(*E*_*F*_) is the density of states (DOS) near the Fermi level, *λ* is the electron–boson coupling strength, and μ^*^ is the Coulomb pseudopotential that describes the residual Coulomb repulsion between quasiparticles^2^. For simplicity, we assume that all of the non-isotropic **q**- and **k**-dependencies that appear in a more realistic formulation of Cooper pairing have been averaged away. Note that within the range of validity of Eq. (1)—viz., 1   ≫ *λ* > μ^*^—increasing *λ* (increasing μ^*^) generally enhances (suppresses) *T*_*c*_, respectively, assuming that superconductivity remains the dominant instability.

Experimental methods that boost *T*_*c*_ are highly desired from a practical perspective. Furthermore, by analyzing how these available knobs couple to the normal-state properties on the right side of Eq. (1), one can envisage engineering the electronic structure and electron–boson coupling to optimize *T*_*c*_. For example, increasing *N*(*E*_*F*_) is a frequently suggested route towards realizing higher *T*_*c*_, but how to achieve this for specific materials often remains unclear.

Historically, chemical doping and hydrostatic pressure have been the most common knobs used to manipulate superconductivity. Unfortunately, doping has the complication of explicitly introducing substitutional disorder, whereas pressure studies are incompatible with most probes of electronic structure. Moreover, because large pressures are usually required to appreciably increase *T*_*c*_^3^, pressure-enhanced superconductivity exists transiently—oftentimes in different structural polymorphs than at ambient conditions—rendering it inaccessible for applications.

An alternative strategy for controlling superconductivity is epitaxial strain engineering. This approach is static, disorder-free, allows for the use of sophisticated experimental probes^4^, and enables integration with other materials in novel artificial interfaces and device structures^5,6^. To date, epitaxial strain has only been used to modulate *T*_*c*_ in known superconductors^7–12^. In this article, we describe the creation of a new superconductor through epitaxial strain, starting from a compound, RuO_2_, previously not known to be superconducting. By comparing the results of angle-resolved photoemission spectroscopy (ARPES) experiments with density functional theory (DFT) calculations, we show that splittings between the effective low-energy *d* orbital degrees of freedom in RuO_2_ respond sensitively to appropriate modes of strain, and we discuss how this approach may open the door to strain tuning of superconductivity in other materials.

## Results

### Electrical and structural characterization of RuO_2_ thin films

**Fig. 1:**
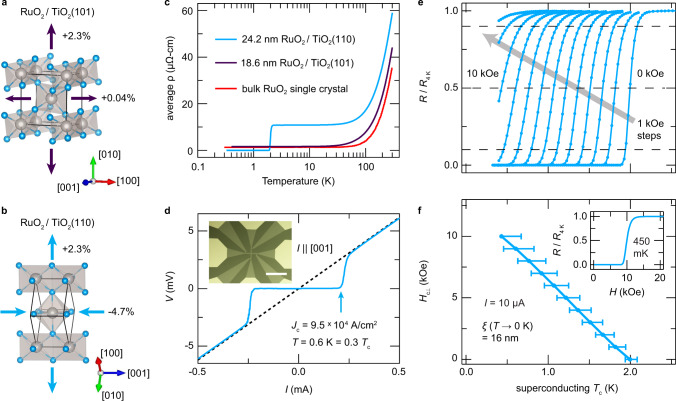
Electrical transport behavior of bulk RuO_2_ single crystals and epitaxially strained RuO_2_ thin films. **a, b** Schematic diagrams of the crystal structures and in-plane lattice mismatches with TiO_2_ substrates of RuO_2_ thin films synthesized in (101)- and (110)-orientations. Gray and blue spheres represent Ru and O atoms, respectively. **c** Average resistivity versus temperature curves for 24.2 nm thick RuO_2_(110) and 18.6 nm thick RuO_2_(101) films, compared to results for bulk RuO_2_ single crystals from Ref. ^15^. For clarity the bulk RuO_2_ data have been rigidly shifted upward by 1 μΩ-cm (*ρ*_0_  ≈  0.3  μΩ-cm). **d** *V*(*I*) curve measured at 0.6 K on a 10 μm-wide resistivity bridge lithographically patterned on the RuO_2_(110) sample from (**c**) (as shown in the inset: scale bar  = 200  μm), which has the direction of current flow parallel to [001]_rutile_. Similarly large critical current densities *J*_*c*_ are obtained with $I| | [1\overline{1}0]$ (Supplementary Note 1 and Supplementary Fig. 1). **e, f** Upper critical magnetic fields *H*_*c*⊥_ versus superconducting *T*_*c*_s extracted from magnetoresistance measurements for the RuO_2_(110) sample in (**c**) along with a characteristic *R*(*H*) sweep acquired at 0.45 K (inset in (**f**)). Superconducting *T*_*c*_s are taken as the temperatures at which the resistance crosses 50% of its residual normal-state value *R*_ 4 K_; error bars on these *T*_*c*_s indicate where *R* crosses the 90% and 10% thresholds of *R*_4 K_, respectively (cf. the horizontal dashed lines in (**e**)).

Bulk RuO_2_ crystallizes in the ideal tetragonal rutile structure (space group *#*136, *P*4_2_/*mnm*) with lattice constants at 295 K of (*a* = 4.492 Å, *c* = 3.106 Å)^13^. RuO_2_ thin films in distinct epitaxial strain states were synthesized using oxide molecular-beam epitaxy (MBE) by employing different orientations of isostructural TiO_2_ substrates, (*a* = 4.594 Å, *c* = 2.959 Å)^14^. As shown in Fig. 1a, b, the surfaces of (101)-oriented substrates are spanned by the $[\overline{1}01]$ and [010] lattice vectors of TiO_2_, which ideally impart in-plane tensile strains on RuO_2_ (at 295 K) of  +0.04% and  +2.3%, respectively. On TiO_2_(110), the lattice mismatches with RuO_2_ are larger:  −4.7% along [001] and  +2.3% along $[1\overline{1}0]$.

  Figure 1c shows electrical resistivity *ρ*(*T*) measurements for RuO_2_ films, along with results for bulk RuO_2_ single crystals from Ref. ^15^. To compare with bulk, for the thin-film samples we plot the geometric mean of the components of *ρ* along the two in-plane directions; the intrinsic resistive anisotropy is known to be small^16^, consistent with our findings (Supplementary Note 1 and Supplementary Fig. 1). *ρ*(*T*) data for the lightly strained RuO_2_/TiO_2_(101) sample—henceforth referred to as RuO_2_(101)—are nearly indistinguishable from bulk, exhibiting metallic behavior with a low residual resistivity *ρ*(0.4 K) < 2  μΩ-cm. In contrast, a clear superconducting transition is observed for the more heavily strained RuO_2_/TiO_2_(110) sample—referred to as RuO_2_(110)—at *T*_*c*_ = 2.0 ± 0.1  K.

Magnetoresistance measurements (Fig. 1e, f) with *H*_⊥_ applied along [110] (the out-of-plane direction) show a monotonic suppression of *T*_*c*_ with increasing fields and an extrapolated value of *H*_*c*⊥_(*T* → 0 K) = 13.3 ± 1.5 kOe, corresponding to an average in-plane superconducting coherence length of *ξ*(*T* → 0 K) = 15.8 ± 0.9 nm (Supplementary Note 2 and Supplementary Fig. 2). In Fig. 1d, we show a *V*(*I*) curve measured on a lithographically patterned resistivity bridge at *T*/*T*_*c*_ = 0.3, from which we extract a critical current density *J*_*c*_ = (9.5 ± 1.2) × 10^4^ A/cm^2^. This large value of *J*_*c*_ (over one order of magnitude larger than values reported on typical elemental superconductors with comparable *T*_*c*_s) indicates that the superconductivity in RuO_2_(110) does not arise from a filamentary network, structural defects, minority phases, or from the substrate–film interface, which would all yield much smaller values of *J*_*c*_.

**Fig. 2:**
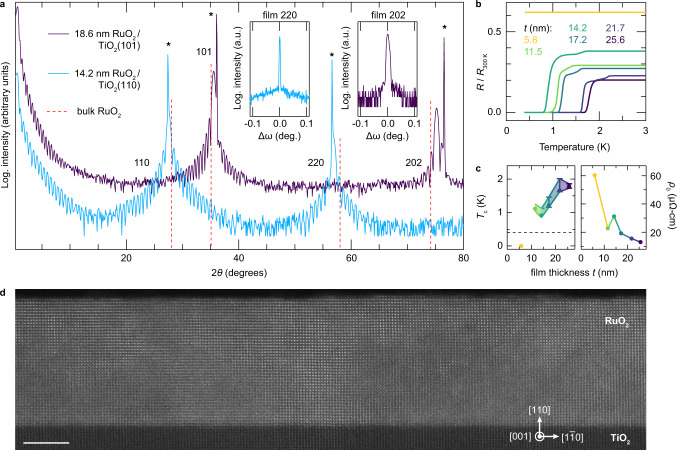
Structural characterization of epitaxially strained RuO_2_ thin films, and film-thickness-dependent superconductivity for RuO_2_(110). **a** XRD data acquired with Cu-K*α* radiation along the specular crystal truncation rods for 18.6 nm thick RuO_2_(101) and 14.2 nm thick RuO_2_(110) films. Bragg peaks arising from the TiO_2_ substrates are marked with asterisks, and the peak positions that would be expected for unstrained bulk RuO_2_ are indicated by dashed red lines^13^. Insets display rocking curves with FWHMs  < 0.01° acquired at the 2*θ* values corresponding to the primary 220 and 202 film peaks. Here *q*_∣∣_ is aligned with TiO${\,}_{2}[1\overline{1}0]$ for the (110)-oriented sample, and with TiO${\,}_{2}[\overline{1}01]$ for the (101)-oriented sample. **b** Resistance versus temperature curves for RuO_2_(110) samples with different film thicknesses *t*, normalized to their values at 300 K. **c** Superconducting *T*_*c*_s and residual resistivities *ρ*_0_ plotted versus film thickness for the RuO_2_(110) samples from (**b**). Error bars on *T*_*c*_s have the same meaning as in Fig. 1. The horizontal dashed line represents the base temperature attainable in our refrigerator, 0.4 K. **d** STEM image of the same 14.2 nm thick RuO_2_(110) sample from (**a**–**c**) (scale bar = 5 nm). More comprehensive structural and electrical characterization of the samples shown here are included in Supplementary Notes 3, 4 and Supplementary Figs. 3–10.

In order to disentangle the effects of strain from other possible sources of superconductivity, we compare RuO_2_ films as functions of strain and film thickness, *t*. In Fig. 2a, we plot x-ray diffraction (XRD) data from similar-thickness films of RuO_2_(101) and RuO_2_(110), showing that the bulk-averaged crystal structures of the films are strained as expected along the out-of-plane direction based on their net in-plane lattice mismatches with TiO_2_. The primary 101 and 202 film peaks of RuO_2_(101) are shifted to larger angles than bulk RuO_2_, corresponding to a 1.1% compression of *d*_101_, while Nelson-Riley analysis of the primary 110, 220, and 330 (see, e.g., Supplementary Fig. 4) peak positions for RuO_2_(110) evidence a 2.0% expansion of *d*_110_ relative to bulk. In Fig. 2b, c, we plot resistivity data showing that reducing *t* in RuO_2_(110) decreases *T*_*c*_, as is commonly observed in numerous families of thin-film superconductors^17,18^, with *T*_*c*_ dropping below our experimental threshold (0.4 K) between *t* = 11.5 and 5.8 nm. This suppression of *T*_*c*_ with thickness indicates superconductivity is not confined near the substrate–film interface, so possible interfacial modifications of the crystal structure^19^, carrier density^20^, substrate–film mode coupling^21^, and non-stoichiometry in the films or substrates^22–24^ can all be eliminated as potential causes of superconductivity. These conclusions are also supported by the facts that superconductivity is not observed in RuO_2_(101) films, nor in bare TiO_2_ substrates treated in an identical fashion to the RuO_2_ films. Finally, in Fig. 2d we include a scanning transmission electron microscopy (STEM) image of a superconducting RuO_2_(110) sample, which confirms uniform growth of the film over lateral length scales exceeding those expected to be relevant for superconductivity (e.g., *ξ*), and shows a chemically abrupt interface between RuO_2_ and TiO_2_ (Supplementary Fig. 5), with no evidence of minority phases.

We believe the thickness dependence of *T*_*c*_ results primarily from the competition between: (i) an intrinsic strain-induced enhancement of *T*_*c*_ that should be maximized for thinner, commensurately strained RuO_2_(110) films, versus (ii) disorder-induced suppressions of *T*_*c*_ that become amplified in the ultrathin limit (see, e.g., *ρ*_0_ versus *t* in Fig. 2c). While the thinnest films experience the largest substrate-imposed strains, stronger disorder scattering (likely from interfacial defects) reduces *T*_*c*_ below our detection threshold. Films of intermediate thickness (*t*  ≈  10–30 nm) have lower residual resistivities and higher *T*_*c*_s, but do exhibit signatures of partial strain relaxation. Nevertheless, a detailed analysis of misfit dislocations by STEM and XRD reciprocal-space mapping (Supplementary Notes 3, 4 and Supplementary Figs. 8–10) indicates that these films are largely structurally homogeneous and, on average, much closer to commensurately strained than fully relaxed. Finally, in much thicker samples (e.g., *t*  =  48 nm) where a more significant volume fraction of the film should be relaxed, the strain is further released by oriented micro-cracks that make such samples spatially inhomogeneous and cause severely anisotropic distributions of current flow, preventing reliable resistivity measurements (Supplementary Fig. 11).

### DFT calculations and ARPES measurements

**Fig. 3:**
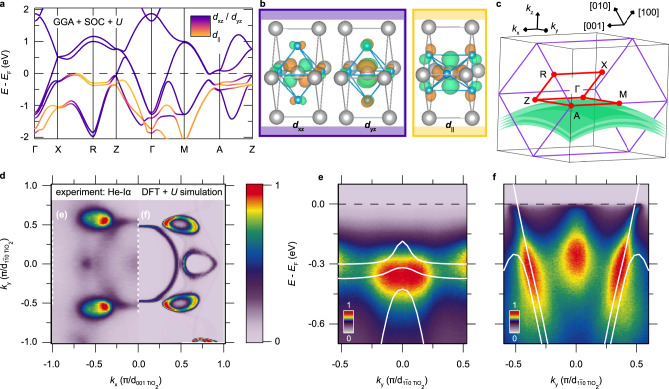
Electronic structure of RuO_2_. **a** Non-magnetic band structure of RuO_2_(110) according to DFT, calculated within the generalized gradient approximation (GGA) including spin–orbit coupling (SOC) and a static  + *U*  =  2  eV correction on the Ru sites. The color scale indicates the magnitudes of projections of the Kohn-Sham eigenstates at each **k** onto Ru-centered Wannier functions with *d*_∣∣_ and (*d*_xz_, *d*_yz_) orbital characters, which are constructed from the manifold of self-consistent eigenstates spanning *E*_*F*_ and are plotted in drawings of the crystal structure in (**b**). Ru (O) atoms are colored gray (blue), as in Fig. 1a, b. Green and orange surfaces in (**b**) represent isosurfaces of the Wannier functions that have equal absolute magnitudes, but opposite (i.e., positive and negative) signs, respectively. **c** Brillouin zone schematic defining the coordinate system utilized for describing ARPES measurements of the electronic structure on (110)-oriented surfaces: *k*_*x*_ ∣∣ [001]_rutile_, ${k}_{y}\ | | \ {[1\overline{1}0]}_{{\rm{rutile}}}$, and *k*_*z*_ ∣∣ [110]_rutile_. The Brillouin zone of the parent tetragonal rutile structure is outlined in purple, the high-symmetry contour for the spaghetti plot from (**a**) is colored red, and the region probed on (110)-oriented surfaces with He-I*α* photons (21.2 eV) is shaded green (Supplementary Note 6 and Supplementary Fig. 13). **d** Slice through the Fermi surface experimentally measured for a 7 nm thick RuO_2_(110) film (left), compared to the Fermi surface from DFT + *U* simulations (right) projected onto the region of the Brillouin zone colored green in (**c**). *E*(*k*) spectra acquired along the one-dimensional cuts indicated by dashed white lines in (**d**) show: **e** flat bands with *d*_∣∣_ orbital character and **f** more dispersive bands with (*d*_xz_, *d*_yz_) character, both consistent with DFT + *U* expectations (solid white lines). The intensities of the experimental data shown in (**d**–**f**) and of the DFT simulations shown in (**d**) are plotted in arbitrary units where we define 0 (1) to be the minimum (maximum) value, respectively, of the given data set. Only relative changes in intensity within a given panel (as visualized by the false color scales) are meaningful.

Having established the strain-induced nature of the superconductivity in RuO_2_(110), we now explore its underlying origin using a combination of DFT and ARPES. In Fig. 3a, we present the electronic structure of commensurately strained RuO_2_(110) calculated by DFT + *U* (*U* = 2  eV), following the methods of Berlijn et al.^13^. Despite being constructed of RuO_6_ octahedra having the same 4*d*^4^ electronic configuration as in (Ca,Sr,Ba)RuO_3_, the electronic structure of RuO_2_ is markedly different from that of perovskite-based ruthenates. These distinctions arise from a sizable ligand-field splitting of the *t*_2*g*_ orbitals, such that the most natural description of the low-energy electronic structure is in terms of states derived from two distinct types of orbitals: *d*_∣∣_ and (*d*_xz_, *d*_yz_), as illustrated by plots of Wannier functions in Fig. 3b^25,26^. Viewed in the band basis in Fig. 3a, the differentiation in **k**-space between these orbitals becomes apparent: the near-*E*_*F*_* d*_∣∣_ states (yellow-orange) form mostly flat bands concentrated around the *k*_001_ = *π*/*c* (i.e., Z-R-A) plane, whereas the (*d*_xz_, *d*_yz_) states (purple) form more isotropically dispersing bands distributed uniformly throughout the Brillouin zone.

In many other *d*^4^ ruthenates (such as Sr_2_RuO_4_ and Ca_2_RuO_4_), static mean-field electronic structure calculations (such as DFT + *U*) often predict quantitatively incorrect effective masses^27–31^—and sometimes even qualitatively incorrect ground states^32^—because these approaches neglect local (atomic-like) dynamical spin-orbital correlations (driven by Hund’s rules) that strongly renormalize the low-energy quasiparticle excitations. Therefore, it is imperative to compare DFT calculations for RuO_2_ with experimental data, to establish the reliability of any theoretically predicted dependence of the electronic structure on strain. The left half of Fig. 3d shows the Fermi surface of RuO_2_(110) measured with He-I*α* (21.2  eV) photons at 17 K, which agrees well with a non-magnetic DFT + *U* simulation of the Fermi surface at a reduced out-of-plane momentum of *k*_110_ = −0.2 ± 0.2 *π*/*d*_110_ (right half of Fig. 3d). In Fig. 3e, f, we plot energy versus momentum spectra acquired along the white dashed lines in Fig. 3d: in Fig. 3e, the spectrum is dominated by the flat *d*_∣∣_ bands centered around a binding energy of 300 meV, whereas in Fig. 3f the (*d*_xz_, *d*_yz_)-derived bands are steeply dispersing and can be tracked down to several hundred meV below *E*_*F*_, both of which are well reproduced by DFT + *U* calculations. The reasonable agreement between the experimentally measured and DFT band velocities is consistent with recent ARPES studies of Ir-doped RuO_2_ single crystals^33^ and with earlier specific heat measurements of the Sommerfeld coefficient in bulk RuO_2_, which suggested a modest momentum-averaged quasiparticle mass renormalization of *γ*_exp._ = 1.45*γ*_DFT_^34,35^. The fact that the true electronic structure of RuO_2_ can be well accounted for by DFT + *U* allows us to utilize such calculations to understand how epitaxial strains can be employed to engineer features of the electronic structure to enhance the instability towards superconductivity.

### Evolution of electronic structure under strain

**Fig. 4:**
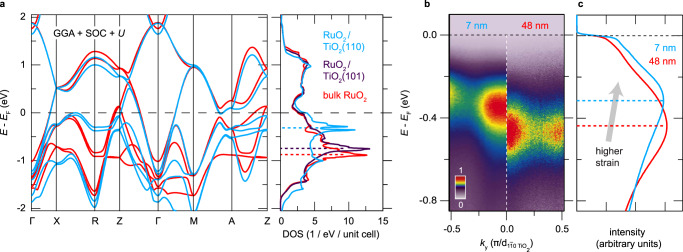
Strain-induced changes to the electronic structure of RuO_2_. **a** DFT + *U* (*U* = 2 eV) band structures and corresponding density of states (DOS) traces for bulk RuO_2_ and epitaxially strained RuO_2_(110) and RuO_2_(101) thin films. The RuO_2_(101) results are omitted from the spaghetti plot for clarity since they are very similar to bulk. **b** Comparison of *E*(*k*) spectra along the cut shown in Fig. 3e for two different RuO_2_(110) samples: a highly strained 7 nm thick film (left), and a partially strain-relaxed 48 nm thick film (right). The false color scale used to visualize the intensities in each spectrum is defined and normalized in the same way as in Fig. 3. **c** As an approximate proxy of the total DOS, for these samples we plot the energy distribution curves of photoemission intensity averaged over the entire region of **k**-space probed experimentally with 21.2 eV photons (cf. Fig. 3c), which demonstrate that the epitaxial strains imposed by TiO_2_(110) substrates shift *d*_∣∣_ states towards *E*_*F*_ and thereby increase *N*(*E*_*F*_).

In Fig. 4a, we show the strain dependence of the DFT-computed band structure and DOS for RuO_2_(110), RuO_2_(101), and bulk RuO_2_. While the results for RuO_2_(101) are almost identical to bulk, the results for RuO_2_(110) exhibit significant differences: the large *d*_∣∣_-derived peak in the DOS (centered around a binding energy of 800 meV for bulk) is split into multiple peaks for RuO_2_(110), several of which are shifted closer to the Fermi level, thereby increasing *N*(*E*_*F*_). In our studies, we found that this strain-dependent trend was robust against details of the DFT calculations, such as whether *U* was finite (Supplementary Note 5 and Supplementary Fig. 12). In order to determine whether this strain dependence of *N*(*E*_*F*_) is realized in experiment, we compared the electronic structure of a thin (7 nm) highly strained RuO_2_(110) film with a much thicker (48  nm) partially strain-relaxed RuO_2_(110) film. The surface lattice constants of the 48 nm thick film were closer to bulk RuO_2_ than the 7 nm thick film (Supplementary Note 7 and Supplementary Fig. 14), so we expect that the surface electronic structure probed by ARPES of the thicker film to be more representative of bulk RuO_2_. Comparisons between the RuO_2_(110) and RuO_2_(101) surfaces are less straightforward, since different parts of the three-dimensional Brillouin zone are sampled by ARPES (Supplementary Note 8 and Supplementary Fig. 15). Figure 4b shows *E*(*k*) spectra side by side for the 7 nm (left) and 48 nm (right) films of RuO_2_(110) along the same cut through **k**-space from Fig. 3e where the photoemission intensity is dominated by *d*_∣∣_ initial states. The higher levels of strain present at the film surface for the 7 nm thick sample cause a substantial shift of the flat bands towards *E*_*F*_ by 120 ± 20 meV relative to the more strain-relaxed 48 nm thick sample. Integrating the ARPES data over the full measured region of **k**-space for both samples gives the average energy distribution curves plotted in Fig. 4c, which show that spectral weight near *E*_*F*_ is enhanced as the *d*_∣∣_ states move towards *E*_*F*_, in qualitative agreement with the trend predicted by DFT. Our results indicate that the primary electronic effect of the epitaxial strains in RuO_2_(110) is to alter the relative occupancies of the *d*_∣∣_ and (*d*_xz_, *d*_yz_) orbitals as compared with bulk, and to push a large number of states with *d*_∣∣_ character closer to *E*_*F*_, which enhances *N*(*E*_*F*_) and likely *T*_*c*_.

## Discussion

Observations of Fermi-liquid-like quasiparticles near *E*_*F*_^34,36–38^ that scatter at higher energies primarily via their interaction with phonons^16,35^, along with the fact that superconductivity in RuO_2_(110) persists in the dirty limit (Supplementary Note 4 and Supplementary Fig. 9), are both consistent with conventional Cooper pairing, suggesting that calculations assuming an electron–phonon mechanism may be enlightening. We performed DFT-based Migdal-Eliashberg calculations of *T*_*c*_ for bulk RuO_2_ and commensurately strained RuO_2_(110) that indeed indicate epitaxial strain can enhance *T*_*c*_ by several orders of magnitude. For bulk RuO_2_, we find that the empirical Coulomb pseudopotential must satisfy μ^*^ > 0.30 to be compatible with the experimentally measured least upper bound on *T*_*c*_ (*T*_*c*_ < 0.3  K^15^). For this range of μ^*^, *T*_*c*_ for RuO_2_(110) can be as high as 7 K (Supplementary Note 9 and Supplementary Fig. 16). A robust strain-induced enhancement of the electron–phonon coupling *λ*_el−ph_ boosts *T*_*c*_ by a factor of 20 (for μ^*^ = 0.30), and this ratio becomes even larger for higher values of μ^*^—e.g., for μ^*^ = 0.37, *T*_*c*_(110)/*T*_*c*_(bulk) = 5 K/5 mK). Although these estimations of *T*_*c*_ are broadly consistent with our experimental findings, conventional superconductivity in RuO_2_ remains a working hypothesis until measurements of the order parameter are possible.

In principle, assuming that all Fermi liquids are eventually unstable towards some channel(s) of Cooper pairing at sufficiently low temperatures and magnetic fields (including internal fields arising from magnetic impurities), the strain-stabilized superconductivity observed here in RuO_2_ is not strictly a change in the ground state of the system. For our purposes, however, extremely low temperatures and fields below what are experimentally achievable can be regarded as effectively zero, justifying our use of phrases such as strain-induced superconductivity interchangeably with huge enhancement of critical temperature. If we limit the scope of this semantic discussion to conventional, non-sign-changing (*s*-wave) order parameters, we note that in the presence of Coulomb repulsion and other effects, an instability towards *s*-wave superconductivity is not present in every system; the electron–phonon coupling generally must exceed some finite critical value. In the present context, the effects of strain reported in this article might be boosting the electron–phonon coupling above the critical value appropriate for RuO_2_, thus inducing a new *s*-wave state that is absent (even in theory) for the unstrained material.

We believe our results demonstrate that a promising strategy to create new transition-metal superconductors is to apply judiciously chosen anisotropic strains that modulate degeneracies among *d* orbitals near *E*_*F*_. Many classic studies of conventional superconductors that have nearly-free-electron states spanning *E*_*F*_ derived from (*s*, *p*) orbitals actually show decreases in *T*_*c*_ under hydrostatic pressure^39^, due to lattice stiffening dominating over any pressure-induced changes to the Hopfield parameter^40^. In a limited number of elemental metals where *T*_*c*_ monotonically increases under pressure (such as vanadium^41^), pressure-induced electron transfer between *s* → *d* orbitals has been suggested as a likely cause of the enhanced transition temperatures^3^; a drawback of this approach, however, is that large pressures of  ⪸ 10  GPa are typically required to, e.g., double *T*_*c*_. More recently, measurements on single crystals of the unconventional superconductor Sr_2_RuO_4_ have shown that appropriately oriented uniaxial pressures of only  ≈1 GPa can boost *T*_*c*_ by more than a factor of two^42^. Independent of the underlying mechanism, it appears that anisotropic strains may prove to be significantly more efficacious than hydrostatic pressure for tuning superconductivity in multi-orbital systems, as shown here for RuO_2_, as well as in Sr_2_RuO_4_.

Sizable coupling between the lattice and electronic degrees of freedom in rutile-like crystal structures has been well established both theoretically^26^ and experimentally in VO_2_, where strain-induced variations in the orbital occupancies can be used to modify the metal-insulator transition temperature by *δT*_MIT_ ≈ 70 K^43,44^. Therefore, it may be promising to explore other less strongly correlated (i.e., 4*d* and 5*d*) rutile compounds such as MoO_2_ for strain-stabilized superconductivity, instead of employing chemical doping^45–47^. Finally, since RuO_2_/TiO_2_(110) is the first known stoichiometric superconductor within the rutile family, further optimization of the superconductivity may enable the creation of structures that integrate superconductivity with other functional properties that have been extensively studied in other rutile compounds, such as high photocatalytic efficiency, half-metallic ferromagnetism, and large spin Hall conductivities.

## Methods

### Film synthesis

Epitaxial thin films of RuO_2_ were synthesized on various orientations of rutile TiO_2_ substrates using a GEN10 reactive oxide MBE system (Veeco Instruments). Prior to growth, TiO_2_ substrates (Crystec, GmbH) were cleaned with organic solvents, etched in acid, and annealed in air to produce starting surfaces with step-terrace morphology, following the methods in Ref. ^48^. Elemental ruthenium (99.99% purity, ESPI Metals) was evaporated using an electron-beam evaporator in background oxidant partial pressures of 1 × 10^−6^ − 5 × 10^−6^  Torr of distilled ozone (≈80% O_3_ + 20% O_2_) at substrate temperatures of 250–400 °C, as measured by a thermocouple. Reflection high-energy electron diffraction was used to monitor the surface crystallinity of the films in situ and showed characteristic oscillations in intensity during most of the Ru deposition, indicating a layer-by-layer growth mode following the initial nucleation of several-monolayer-thick RuO_2_ islands^49^.

### Film characterization

The crystal structures of all RuO_2_ thin-film samples were characterized via lab-based x-ray diffraction (XRD) measurements with Cu-K*α* radiation (Rigaku SmartLab and Malvern Panalytical Empyrean diffractometers). Four-point-probe electrical transport measurements were conducted from 300 K down to a base temperature of 0.4 K using a Physical Properties Measurement System equipped with a He-3 refrigerator (Quantum Design). All RuO_2_/TiO_2_(110) samples were superconducting with *T*_*c*_s ranging from 0.5 to  2.4 K, except for ultrathin films with residual resistivities *ρ*_0_ ⪸  40 μΩ-cm, as shown in Fig. 2 and Supplementary Fig. 9.

A subset of films studied by XRD and transport were also characterized in situ by ARPES and low-energy electron diffraction (LEED). For these measurements, films were transferred under ultrahigh vacuum immediately following growth to an analysis chamber with a base pressure of 5 × 10^−11^ Torr equipped with a helium plasma discharge lamp, a hemispherical electron analyzer (VG Scienta R4000), and a four-grid LEED optics (SPECS ErLEED 150).

A subset of films studied by XRD and transport were also imaged using cross-sectional STEM. Cross-sectional specimens were prepared using the standard focused ion beam (FIB) lift-out process on a Thermo Scientific Helios G4 X FIB. High-angle annular dark-field STEM (HAADF-STEM) images were acquired on an aberration-corrected FEI Titan Themis at 300 keV with a probe convergence semi-angle of 21.4 mrad and inner and outer collection angles of 68 and 340  mrad.

### Electronic structure calculations

Non-magnetic DFT calculations for the electronic structure of RuO_2_ were performed using the Quantum ESPRESSO software package^50,51^ with fully relativistic ultrasoft pseudopotentials for Ru and O^52^. We represented the Kohn-Sham wavefunctions in a basis set of plane waves extending up to a kinetic energy cutoff of 60  Ry, and used a cutoff of 400 Ry for representing the charge density. Brillouin zone integrations were carried out on an 8 × 8 × 12 *k*-mesh with 70 meV of Gaussian smearing. Perdew, Burke, and Ernzerhof’s parametrization of the generalized gradient approximation was employed as the exchange-correlation functional^53^, supplemented by an on-site correction of  +*U*_eff_ = *U* − *J* = 2 eV within spheres surrounding the Ru sites, following Ref. ^13^.

After obtaining self-consistent Kohn-Sham eigenstates via DFT, we used the pw2wannier and Wannier90 codes^54^ to construct 20 Wannier functions spanning the manifold of eigenstates surrounding *E*_*F*_ (20 = 10 *d*-orbitals per Ru atom  ×  2 Ru atoms per unit cell). Following Ref. ^55^, to account for the non-symmorphic space group symmetries of rutile crystal structures, we referenced the trial orbitals employed in the Wannierisation routine to locally rotated coordinate systems centered on the two Ru sites within each unit cell. Orbital designations employed in the main text such as *d*_∣∣_ and (*d*_xz_, *d*_yz_) refer to projections onto this basis of Wannier functions. The more computationally efficient Wannier basis was used to calculate quantities that required dense *k* meshes to be properly converged, such as the projected Fermi surface in Fig. 3d (51 × 51 × 51 *k*-mesh) and the near-*E*_*F*_ density of states traces in Fig. 4a (32 × 32 × 48 *k*-meshes).

Because the RuO_2_ samples studied in this work are thin films subject to biaxial epitaxial strains imposed by differently oriented rutile TiO_2_ substrates, we performed DFT + Wannier calculations of the electronic structure for several different crystal structures of RuO_2_ as described in Supplementary Note 5 and Supplementary Table 1. We used the ISOTROPY software package^56^ to study distortions of the parent tetragonal rutile crystal structure that are induced in biaxially strained thin films. Crystal structures and Wannier functions were visualized using the VESTA software package^57^.

### Electron–phonon coupling calculations

To generate the inputs required for the electron–phonon coupling calculations described below, first-principles electronic structure and phonon calculations were performed using the Quantum ESPRESSO software package with norm-conserving pseudopotentials and plane-wave basis sets^50,51^. Here we employed a kinetic energy cutoff of 160 Ry, an electronic momentum *k*-point mesh of 16 × 16 × 24, 20  meV of Methfessel-Paxton smearing for the occupation of the electronic states, and a tolerance of 10^−10^ eV for the total energy convergence. The generalized gradient approximation as implemented in the PBEsol functional^58^ was employed as the exchange-correlation functional. For the Wannier interpolation, we used an interpolating electron-momentum mesh of 8 × 8 × 12 and a phonon-momentum mesh of 2 × 2 × 3. Results for bulk RuO_2_ were calculated using the crystal structure that minimizes the DFT-computed total energy with the PBEsol functional: (*a* = 4.464 Å, *c* = 3.093 Å) and *x*_oxygen_ = 0.3062. Results for strained RuO_2_(110) were calculated by changing the lattice constants of this simulated bulk crystal structure by  +2.3% along $[1\overline{1}0]$,  −4.7% along [001],  +2.2% along [110], and setting *x*_oxygen_ = *y*_oxygen_ = 0.2996. The lattice parameter along [110] and internal coordinates of this simulated RuO_2_(110) structure were determined by allowing the structure to relax so as to (locally) minimize the DFT-computed total energy.

Electron–phonon coupling calculations were performed using the EPW code^59^, using an interpolated electron-momentum mesh of 32 × 32 × 48 and an interpolated phonon-momentum mesh of 8 × 8 × 12. The isotropic Eliashberg spectral function *α*^2^*F*(*ω*) and total electron–phonon coupling constant *λ*_el−ph_ (integrated over all phonon modes and wavevectors) were calculated with a phonon smearing of 0.2 meV. From the calculated *α*^2^*F*(*ω*) and *λ*_el−ph_, we estimated the superconducting transition temperature using the semi-empirical McMillan-Allen-Dynes formula^60,61^:

2 $${T}_{c}=\frac{{\omega }_{\mathrm{log}\,}}{1.2}\exp \left[-\frac{1.04(1+{\lambda }_{{\rm{el}}-{\rm{ph}}})}{{\lambda }_{{\rm{el}}-{\rm{ph}}}-{\mu }^{* }(1+0.62{\lambda }_{{\rm{el}}-{\rm{ph}}})}\right]$$

## Supplementary information

Supplementary Information

Peer Review File

## Data Availability

The data supporting the findings of this study are available within the paper and supplementary information. Data connected to the study from PARADIM facilities are available at paradim.org. Any additional data connected to the study are available from the corresponding author upon reasonable request.
