# Supplementary material for: Strain-stabilized superconductivity
Source: Nat Commun. 2021 Jan 4;12:59. doi: 10.1038/s41467-020-20252-7 (PMC7782483; doi:10.1038/s41467-020-20252-7)
Supplement: Supplementary file 1 — Supplementary Information [file 41467_2020_20252_MOESM1_ESM.pdf]

Supplementary Information: Strain-stabilized superconductivity

J. P. Ruf,[1] H. Paik,[2] N. J. Schreiber,[3] H. P. Nair,[3] L. Miao,[1] J. K. Kawasaki,[1,4] J. N. Nelson,[1] B. D. Faeth,[1] Y. Lee,[1] B. H. Goodge,[5,6] B. Pamuk,[5] C. J. Fennie,[5] L. F. Kourkoutis,[5,6] D. G. Schlom,[3,6] and K. M. Shen[1,6]

[1]*Department of Physics, Laboratory of Atomic and Solid State Physics, Cornell University, Ithaca, NY 14853, USA*
[2]*Platform for the Accelerated Realization, Analysis, and Discovery of Interface Materials (PARADIM), Cornell University, Ithaca, New York 14853, USA*
[3]*Department of Materials Science and Engineering, Cornell University, Ithaca, NY 14853, USA*
[4]*Department of Materials Science and Engineering, University of Wisconsin, Madison WI 53706*
[5]*School of Applied and Engineering Physics, Cornell University, Ithaca, New York 14853, USA*
[6]*Kavli Institute at Cornell for Nanoscale Science, Ithaca, NY 14853, USA*

Supplementary Note 1: ELECTRICAL TRANSPORT MEASUREMENTS ON PATTERNED RESISTIVITY BRIDGES

In Supplementary Fig. 1, we show how the electrical transport properties of an $RuO_2/TiO_2(110)$ sample depend on the direction of current flow in the film when it is confined to flow along the orthogonal in-plane crystallographic axes, [001] and $[1\bar{1}0]$. Prior to lithographically patterning resistivity bridges on the film, we measured the resistance versus temperature of the entire 10 mm × 10 mm × 24.2 nm thick film by wire bonding four contacts directly to the surface of the sample in an in-line contact geometry. Such a contact geometry probes the geometric mean of the two diagonal components of the in-plane resistivity tensor, i.e. $\sqrt{\rho_{001}\rho_{1\bar{1}0}}$, neglecting small finite-size corrections that depend on how the contacts are oriented relative to the edges of the wafer [1]. The results of these measurements are shown by the blue traces in Supplementary Fig. 1a-b; these are the same data plotted on a logarithmic temperature scale in Fig. 1c of the main text.

Since RuO_2 has a tetragonal crystal structure in bulk (and orthorhombic or perhaps monoclinic in (110)-oriented films), ρ_{001} and $\rho_{1\bar{1}0}$ are not guaranteed by symmetry to be equal. The intrinsic transport anisotropy in bulk RuO_2 is known to be small, with differences between ρ_{100} and ρ_{001} that are less than 10% at 300 K [2, 3]; however, in heteroepitaxial thin films it is common for highly oriented structural defects—e.g., those nucleated at step edges on the substrate—to induce sizable extrinsic anisotropies between the different in-plane components of the resistivity tensor [4, 5]. To investigate this possibility in this work, we used standard lithographic techniques to pattern the same $RuO_2/TiO_2(110)$ sample into four-point resistivity bridges with dimensions 55 μm (length) × 10 μm (width) × 24.2 nm (thickness), where the direction of current flow is confined (via lithography) to be aligned with specific crystallographic directions. In the course of performing the lithography, we noticed that the TiO_2 substrates became mildly conducting, possibly due to oxygen vacancies formed during ion milling, as has been reported to occur for $SrTiO_3$ [6]. Therefore, we annealed the wafer containing the patterned resistivity bridges in air at elevated temperatures until the substrate again read open-circuit two-point resistances (> 100 MΩ); 2 hours at 500° C was found to be sufficient.

The results of electrical measurements on these patterned resistivity bridges are shown by the green and orange traces in Supplementary Fig. 1. The temperature dependence of $\rho(T)$ is qualitatively consistent with the control measurements performed on the entire film before patterning, and the absolute magnitude of the resistivity anisotropies at 300 K and 4 K are both < 20%. Furthermore, the superconducting $\rho(T)$ and $V(I)$ behavior does not depend strongly on the direction of current flow; this is contrary to what would be expected if the superconductivity arose purely from oriented structural defects.

In Supplementary Fig. 1b, we ascribe the substantial decrease in low-temperature resistivities observed in the patterned resistivity bridge data relative to the entire film data to the aforementioned annealing involved in preparing the bridges. We confirmed on other $RuO_2/TiO_2(110)$ samples not containing bridges that post-growth annealing in air generically causes the low-temperature values of ρ to drop, by as much as a factor of four. Because of these complications and additional uncertainties involved in lithographically patterning resistivity bridges on films on TiO_2 substrates, all other electrical transport data presented in the main text and in the supplementary information were acquired by wire bonding directly to the surfaces of as-grown samples that were not subject to any post-growth annealing treatments.

Supplementary Note 2: FITTING AND EXTRAPOLATION OF SUPERCONDUCTING UPPER CRITICAL FIELDS VERSUS TEMPERATURE

In Supplementary Fig. 2, we present the results of magnetoresistance measurements for three $RuO_2/TiO_2(110)$ samples with different film thicknesses; the data in Supplementary Fig. 2a-b are reproduced from Fig. 1e-f of the main text. Each $R(T)$ trace was acquired at a discrete value of the externally applied magnetic field $H_\perp$ (applied perpendicular to the surfaces of the films, along [110]) upon warming the samples up from base temperature

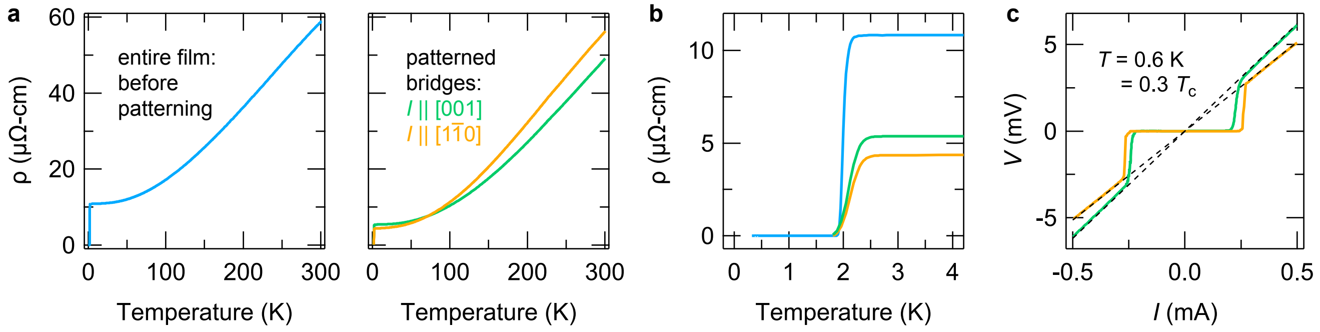

Supplementary Figure 1. **Electrical transport measurements exploring the anisotropy of the resistivity and superconductivity of a 24.2 nm thick $RuO_2/TiO_2(110)$ sample. a - b,** Zero-field $\rho(T)$ data measured on the entire as-grown film (blue) and after lithographically patterning four-point resistivity bridges (green and orange). **c,** Superconducting $V(I)$ curves measured on patterned resistivity bridges with the directions of current flow parallel to [001] and [1$\bar{1}$0].

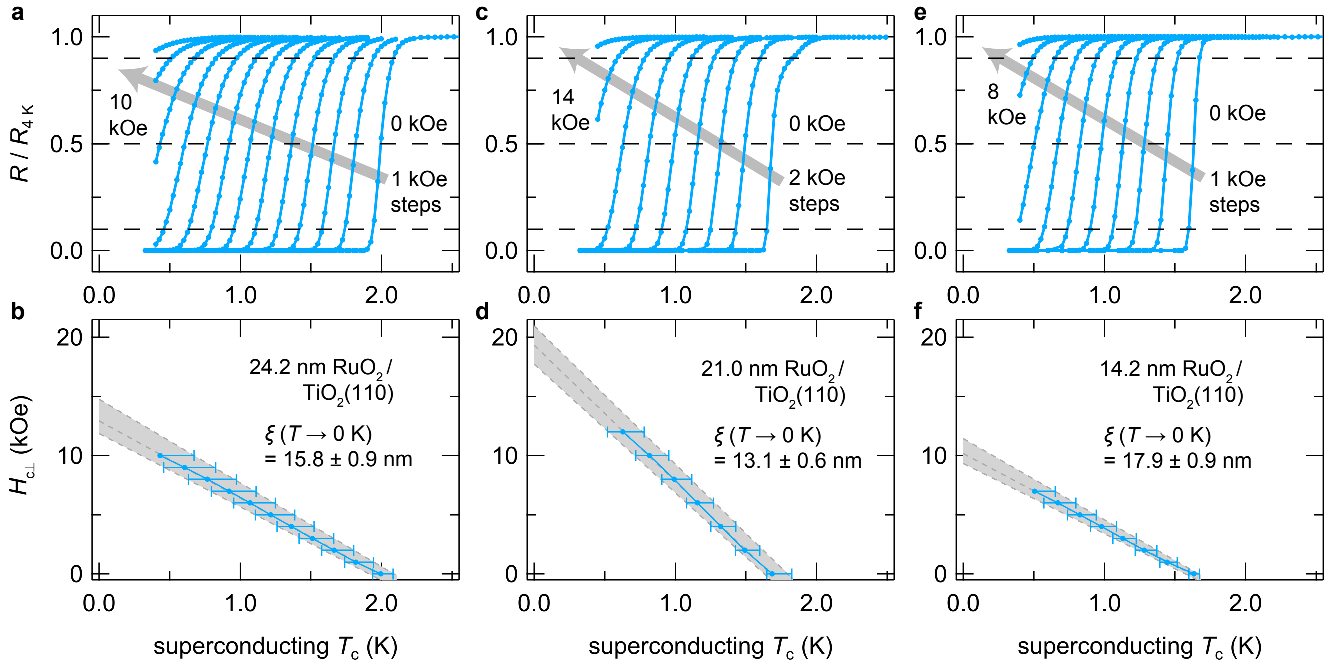

Supplementary Figure 2. **Magnetoresistance measurements for three superconducting RuO_2 /TiO_2(110) samples with different film thicknesses.** Raw data traces in **a, c, e** are normalized to common values $R_{4\,\mathrm{K}} \equiv R(T = 4\,\mathrm{K}, H = 0\,\mathrm{kOe})$ for ease of visualization and analysis. The extracted scaling behavior of the upper critical fields versus superconducting T_cs are plotted in **b, d, f**, along with the superconducting coherence length ξ corresponding to the extrapolated zero-temperature $H_{c\perp}$, cf. Supplementary Eq. (2). T_cs are taken as the temperatures at which R crosses 50% of $R_{4\,\mathrm{K}}$ (middle dashed lines in **a, c, e**); error bars in **b, d, f** indicate the temperatures at which R crosses the 90% and 10% thresholds of $R_{4\,\mathrm{K}}$, respectively (top and bottom dashed lines in **a, c, e**).

through the superconducting transitions. All resistances are normalized to their zero-field values at 4 K, well above the superconducting transitions; since the normal-state $R(T, H)$ behavior of $RuO_2/TiO_2(110)$ in the absence of superconductivity is negligible in this regime of low temperatures and fields, the choice of a single normalization factor $R_{4\,\mathrm{K}}$ for all data does not appreciably affect any of the results that follow. Because percolation effects imply that resistive measurements of critical fields inherently contain some ambiguity about the definition and meaning of $H_{c\perp}$ relative to truly bulk-sensitive measurements of superconductivity [7], here we adopt the same convention employed in the main text: the temperature at which R crosses 50% of $R_{4\,\mathrm{K}}$ is taken as T_c for the given $H_{c\perp}$, and the error bars on the extracted T_c are the temperatures at which R crosses the 90% and 10%

thresholds of $R_{4\text{ K}}$, respectively [8].

While there are considerable quantitative discrepancies in the values of $H_{c\perp}$ and T_c for the different-thickness samples shown in Supplementary Fig. 2, the $H_{c\perp}(T_c)$ scaling behavior is remarkably linear for all samples, with no signs of $H_{c\perp}$ saturation down to reduced temperatures $T/T_c \approx 0.2 - 0.3$, unlike what is expected in, e.g., Werthamer-Helfand-Hohenberg (WHH) theory [9]. For example, evaluating the right hand side of the WHH expression

$$H_{c\perp}(T \to 0\text{ K}) \leq -0.693 \left.\frac{dH_{c\perp}}{dT}\right|_{T=T_c} T_c \qquad (1)$$

places upper bounds of 9.0, 13.5 and 7.1 kOe on $H_{c\perp}(T \to 0\text{ K})$ for these three samples; however, the experimentally measured critical fields at 0.45 K (i.e., $T/T_c = 0.23$, 0.27 and 0.28) are already larger than these bounds: 10.0, 13.7 and 7.4 kOe, respectively. Therefore, to extrapolate $H_{c\perp}$ down to zero temperature, we performed linear Ginzburg-Landau-type fits to all available data and propagated the systematic uncertainties in the definition of $H_{c\perp}$ according to the gray dashed lines. The quoted zero-temperature values of the average in-plane superconducting coherence lengths $\xi(T \to 0\text{ K})$ are obtained from the relation

$$\xi(T \to 0\text{ K}) = \sqrt{\frac{\Phi_0}{2\pi\mu_0 H_{c\perp}(T \to 0\text{ K})}}\ , \qquad (2)$$

where Φ_0 is the superconducting flux quantum and μ_0 is the magnetic permeability of free space.

Notably, these values of $\xi(T \to 0\text{ K})$ are less than values reported for traditional elemental superconductors with comparable T_cs by almost an order of magnitude, corresponding to critical fields that are $\approx 1 - 2$ orders of magnitude greater. While an explanation and understanding of these sizable critical field enhancements are beyond the scope of the present work, they are internally self-consistent with the large critical current densities noted in Fig. 1d of the main text and in Supplementary Fig. 1c. These results may motivate future real-space measurements of the superconducting condensate by scanning-probe techniques. In particular, an interesting question to address is whether the structural defects in $RuO_2(110)$ act as pinning sites for the vortices that form under applied fields, similar to what has been observed in numerous other thin-film superconductors [10], or whether the defects host regions of enhanced superfluid density that effectively act as barriers to vortex motion, akin to twin boundaries in bulk single crystals of iron-based superconductors [11, 12].

Supplementary Note 3: STRUCTURAL AND ELECTRICAL CHARACTERIZATION DATA FOR FILMS SYNTHESIZED ON DIFFERENTLY ORIENTED SUBSTRATES

In Supplementary Fig. 3, we include electrical characterization and more comprehensive lab-based x-ray diffraction (XRD) measurements for the $RuO_2(101)$ and $RuO_2(110)$ films of comparable thickness shown in Fig. 2a of the main text. Supplementary Fig. 3a,e show the zero-field $\rho(T)$ behavior for the two films: the 18.6 nm thick $RuO_2(101)$ film is non-superconducting down to < 0.4 K with a residual resistivity $\rho_0 < 1.7\ \mu\Omega$-cm, whereas the 14.2 nm thick $RuO_2(110)$ film is superconducting at $T_c = 0.92 \pm {}^{0.21}_{0.07}$ K with a residual resistivity $\rho_0 < 32\ \mu\Omega$-cm. Supplementary Fig. 3b,f show rocking curves for the films overlaid on rocking curves for the TiO_2 substrates they were synthesized on: in all cases the coherent components of the film peaks exhibit narrow full width at half maximum (FWHM) values that are limited by the underlying substrate FWHM, as expected for isostructural film growths. In our studies we found that the rocking curve shapes and widths of the TiO_2 substrates supplied by CrysTec, GmbH can vary significantly depending on how the in-plane momentum transfer $q_{||}$ is oriented relative to the crystal axes of a given wafer, which may be due to the Verneuil process used to synthesize the crystals; to give some idea of the magnitude of this asymmetric mosaic spread, we show scans with $q_{||}$ oriented along azimuths separated by 90° for each sample.

In Supplementary Fig. 3c,d and Supplementary Fig. 3g,h we show off-specular $(q_{||}, q_{\perp})$ reciprocal space maps (RSMs) for both samples in regions surrounding HKL Bragg peaks that have $q_{||}$ purely aligned with the crystallographic directions indicated in the labels on the horizontal axes. For reference, the peak positions that would be expected for the lattice parameters of bulk RuO_2 and bulk TiO_2 at 295 K [13, 14] are shown as red and white squares, respectively; the orange squares represent the central peak positions expected for commensurately strained RuO_2 thin films calculated using appropriately constrained density functional theory structural relaxations. To give a more quantitative sense of the logarithmic false color scale used here, the solid white lines overlaid on each plot represent the scattered intensity along the crystal truncation rods (CTRs)—i.e., the one-dimensional line cuts through the RSMs with $q_{||}$ equal to that of the substrate Bragg peaks. These results show that the 18.6 nm thick $RuO_2(101)$ film is coherently strained to the substrate along both in-plane directions, within the $\approx 0.1\%$ resolution of the measurements. The variable widths of the CTRs versus $q_{||}$ in different RSMs are an artifact of instrumental resolution effects—namely, the "tall" incident beam profile convolved with the scattering geometries used to measure each RSM—which we do not attempt to correct for in this work. On the other hand, the 14.2 nm thick $RuO_2(110)$ film is partially

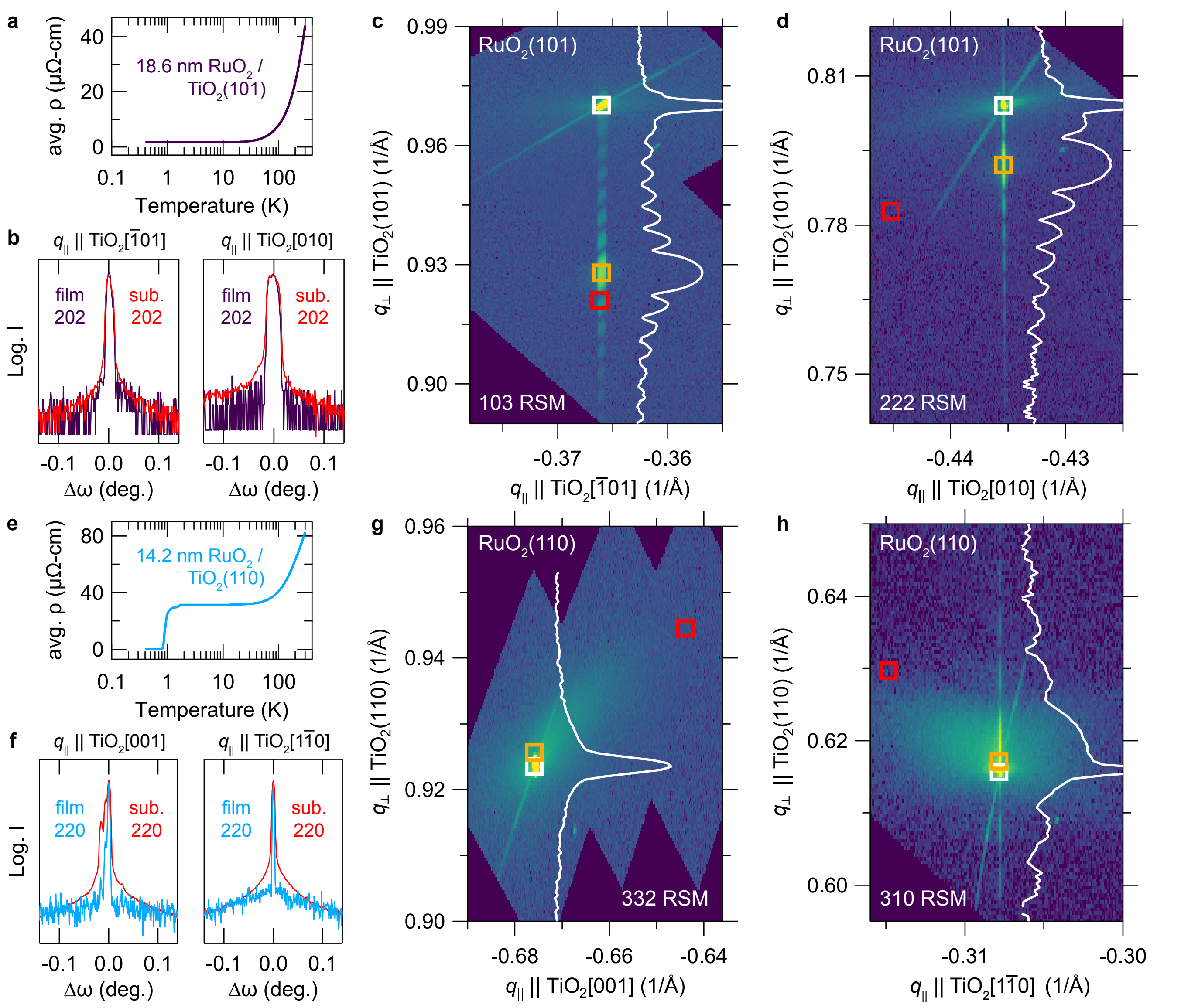

Supplementary Figure 3. **Electrical and structural characterization of (101)- and (110)-oriented RuO_2 thin films.** **a,** Zero-field $\rho(T)$ (geometric mean) data for a non-superconducting 18.6 nm thick $RuO_2(101)$ sample. **b,** Rocking curves for this sample, taken at 2θ values corresponding to the primary film and substrate 202 reflections. The FWHMs are 0.0081° with $q_{||}$ aligned along $[\bar{1}01]$ and 0.021° with $q_{||}$ along [010]. **c - d,** RSMs for this sample near the 103 and 222 reflections. Solid white lines are the scattering profiles along the CTRs. White, red, and orange squares represent the central peak positions expected for bulk TiO_2, bulk RuO_2 and commensurately strained RuO_2 thin films, respectively. The in-plane lattice mismatches of TiO_2 with bulk RuO_2 can be read off directly from the lateral offsets of the white and red squares: +0.04% (tensile) along $[\bar{1}01]$ in **c** and +2.3% along [010] in **d**. **e - h,** Analogous electrical and structural data for a superconducting 14.2 nm thick $RuO_2(110)$ sample. The rocking curve FWHMs at 220 are 0.0042° with $q_{||}$ aligned along [001] (although there are clearly multiple peaks discernible in both the substrate and film curves) and 0.0036° with $q_{||}$ along $[1\bar{1}0]$. RSMs for $RuO_2(110)$ at this film thickness show signatures of partial strain relaxation, because of the larger absolute levels of in-plane lattice mismatch with TiO_2: -4.7% along [001] in **g** and +2.3% in **h**.

strain-relaxed, as evidenced by the more diffuse distribution of scattered intensity versus $q_{||}$ and less prominent finite-thickness fringes versus $q_{\perp}$ along the CTRs. The diminished (or non-existent) contrast between thickness fringes in the CTRs for $RuO_2(110)$ is likely a manifestation of crystalline disorder in the film interplanar spacings, since all (110)-oriented films have abrupt bounding interfaces—cf. the x-ray reflectivity data plotted in Supplementary Fig. 9a.

To further substantiate the partial strain relaxation

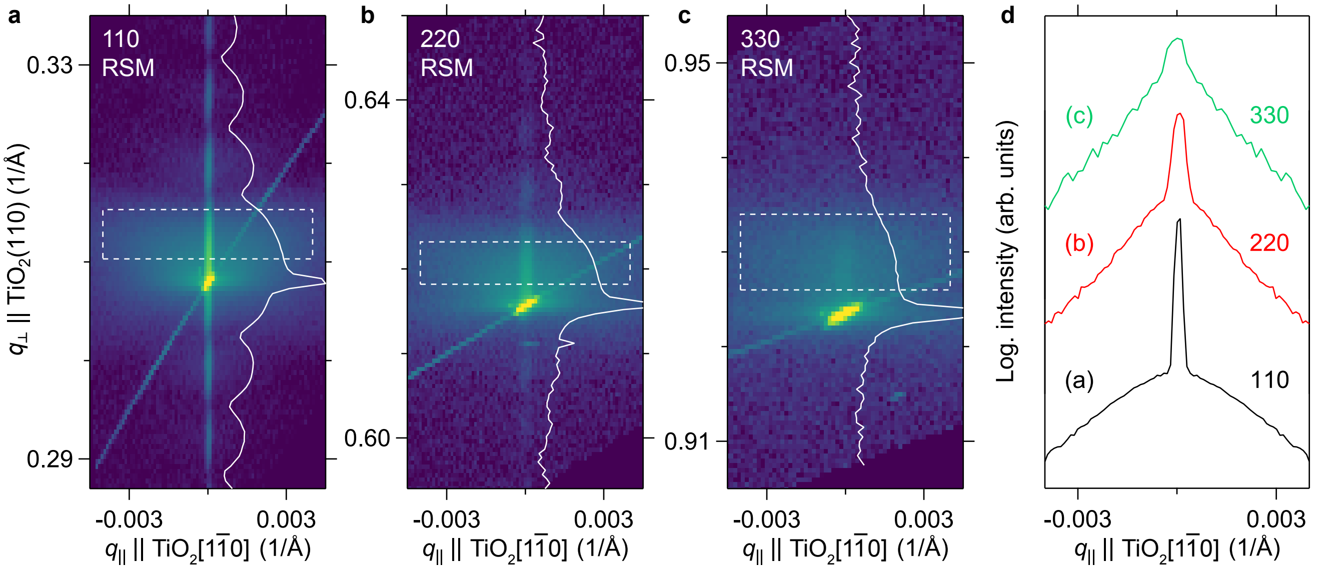

Supplementary Figure 4. **RSMs and rocking curves for a partially strain-relaxed 14.2 nm thick $RuO_2(110)$ sample.** **a - c,** RSMs measured near the 110, 220, and 330 Bragg reflections. Solid white lines are the scattering profiles along the specular ($q_{||} = 0$) CTR. **d,** Line cuts of the intensities averaged over the dashed boxes in **a - c** show rocking curves with a two-component narrow plus broad structure characteristic of partially strain-relaxed epitaxial thin films. The in-plane momentum transfer $q_{||}$ is aligned with $TiO_2[1\bar{1}0]$ in all panels; similar results are obtained with $q_{||}$ along $TiO_2[001]$ (cf. Supplementary Figs. 9c, 10a).

observed in $RuO_2/TiO_2(110)$ samples, we measured RSMs around several Bragg peaks along the specular ($q_{||} = 0$) CTR. Supplementary Fig. 4 summarizes the results of such measurements for the same 14.2 nm thick $RuO_2(110)$ sample for which off-specular RSMs are shown in Supplementary Fig. 3, which was also characterized by XRD and scanning transmission electron microscopy in Fig. 2 of the main text. By taking line cuts averaged over the dashed boxes—which span ranges of $q_\perp$ where the measured intensities are predominantly due to scattering from the film—we obtained the three rocking curves plotted in Supplementary Fig. 4d. Each rocking curve shows a sharp central peak that is resolution-limited in width (or substrate-limited, cf. Supplementary Fig. 3b,f), superimposed on a much broader, nearly Lorentzian (FWHM $= 0.003 - 0.005$ Å^{-1}), component of the scattering that is also centered at $q_{||} = 0$. Furthermore, the integrated intensity of the former coherent component of the scattering decays relative to that of the diffuse component as the magnitude of $|\mathbf{q}| = q_\perp$ increases in progressing from cut (a) to cut (c).

The non-vanishing intensity of the diffuse component in the film rocking curves, and the scaling behavior of how the total integrated intensity is distributed between the coherent and diffuse components as $|\mathbf{q}|$ is varied, are both completely consistent with published data for numerous epitaxial thin films grown on lattice-mismatched substrates where the films are thick enough to exhibit some form of strain relaxation [15–20]. In principle, by analyzing the diffuse scattering profiles around multiple Bragg peaks with $\mathbf{q}$ that project differently onto the Burgers vectors of the relevant misfit dislocations that relax the strain, one can obtain quantitative information on the types of dislocations that exist, the dislocation densities, etc. [21, 22]. We leave a more systematic analysis of this type to future synchrotron XRD studies, where the measurement noise floor is significantly lower and the strongly $\mathbf{q}$-dependent instrumental resolution effects observed here are mitigated by having a more point-like incident beam profile. We note, however, that the similar FWHM values of the diffuse scattering versus $q_{||}$ around the 110, 220, and 330 peaks imply that the structural defects responsible for this scattering are more translational in nature than rotational (which in typical mosaic crystals, produce rocking curves of constant *angular* widths) [15, 16]. Whether the inverses of these FWHM values for the fitted Lorentzians can be directly interpreted as the Fourier transform of a real-space correlation length ($200 - 300$ Å) depends on whether the film is in the limit of *weak (structural) disorder*, in the formalism of Refs. [16, 21].

Supplementary Figs. 5-6 show additional high-angle annular dark-field scanning transmission electron microscopy (HAADF-STEM) data from the same 14.2 nm thick $RuO_2(110)$ sample characterized in Fig. 2 of the main text and in Supplementary Figs. 3-4. Supplementary Fig. 5a shows that the morphology of the RuO_2 film is continuous and epitaxial to the TiO_2 substrate over the largest length scales probed. An intensity line profile taken along the growth direction (yellow line in

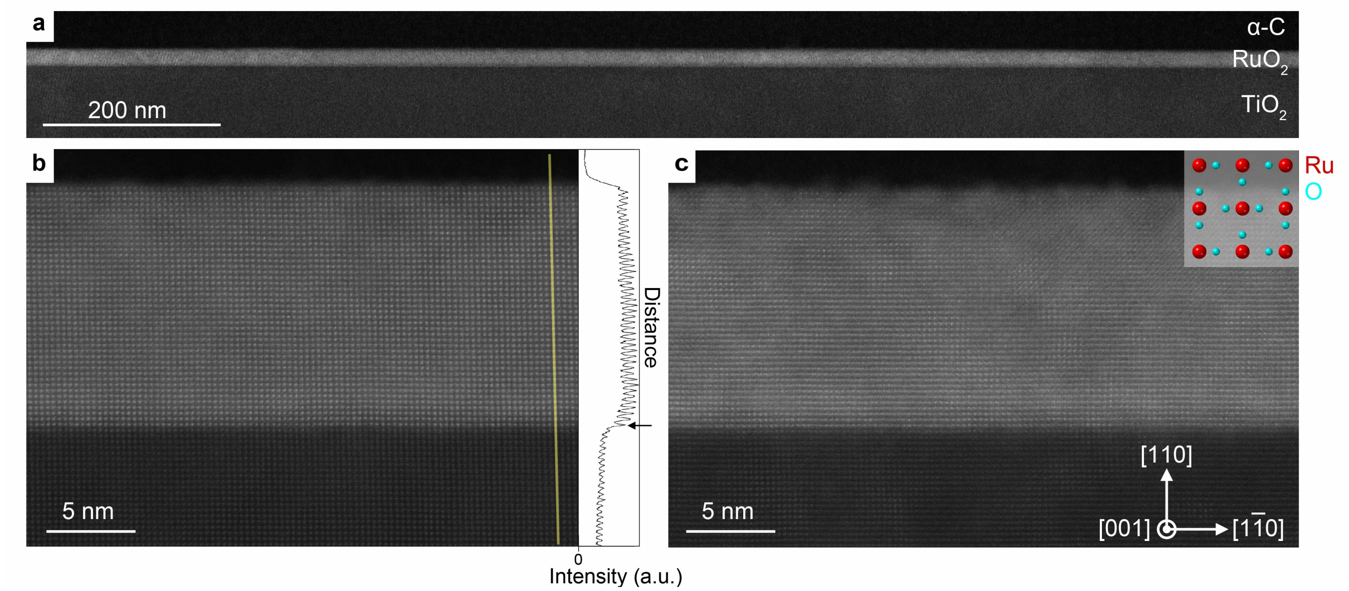

Supplementary Figure 5. **Variation in local crystalline quality across a superconducting 14.2 nm thick RuO_2(110) sample.** **a,** Z-contrast STEM image confirming continuous film growth across a micron-scale field of view. **b,** A crystallographically coherent region of the RuO_2(110) film from **a**. A line cut of the measured intensity across a continuous column of atoms along the growth direction (yellow line) shows an abrupt interface between TiO_2 and RuO_2, indicated by the black arrow. **c,** A relatively less-ordered region of the same film shown in the same projection, perpendicular to $[001]_{\text{rutile}}$. Inset shows the expected structure for this projection (not to scale).

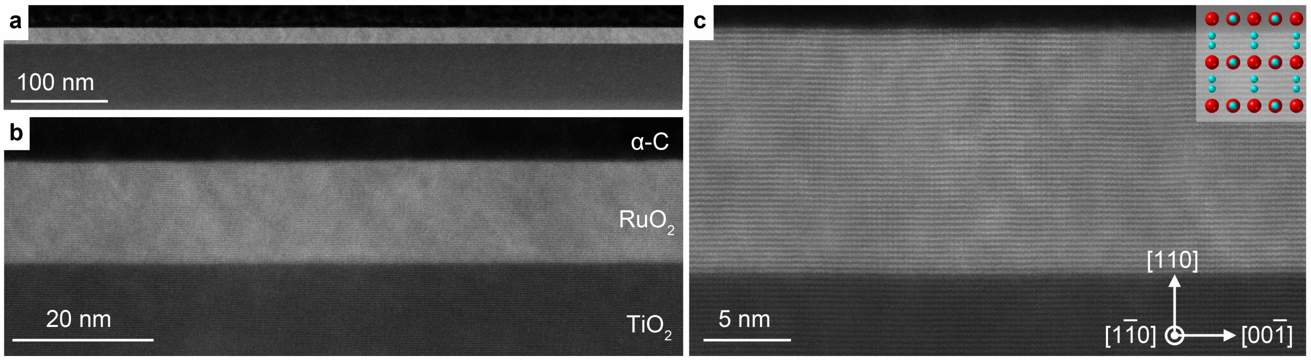

Supplementary Figure 6. **HAADF-STEM structural characterization of a superconducting 14.2 nm thick RuO_2(110) sample in the higher strain direction.** Z-contrast STEM images acquired in [1$\bar{1}$0] projection demonstrate the effects of the -4.7% compressive strain applied by the [001] axis of the TiO_2 substrate. **a,** Continuous film growth is observed across the full length of the STEM lamella, shown here without interruption over several hundreds of nanometers. **b,** Epitaxial film growth, as observed in the orthogonal projection (Supplementary Fig. 5), is again confirmed. **c,** Atomic-resolution image shows the crystalline quality of the strained RuO_2 film. Inset shows the expected structure for this projection (not to scale).

Supplementary Fig. 5b) confirms that an abrupt interface exists between TiO_2 and RuO_2. In particular, a sharp transition from lower intensity peaks in the substrate (Ti: $Z = 22$) to higher intensity peaks in the film (Ru: $Z = 44$) occurs over a region thinner than 1 nm surrounding the black arrow at the substrate-film interface; this indicates that any Ti/Ru chemical interdiffusion is minimal and cannot be the cause of the enhanced superconductivity observed in RuO_2(110). At the lattice scale, we find that different regions from the same film exhibit varying degrees of crystalline coherence under the epitaxial strain applied by the TiO_2 substrate. The lateral in-plane direction imaged in Supplementary Fig. 5 is the [1$\bar{1}$0] axis of the RuO_2 film, subject to +2.3% tensile strain from the TiO_2 substrate. Some regions, such as the one shown in Supplementary Fig. 5b, exhibit ex-

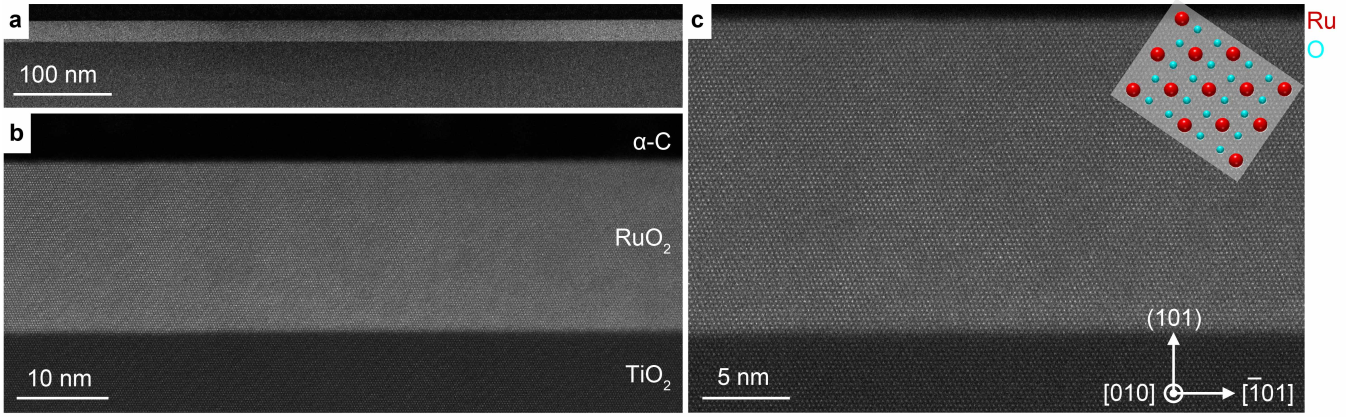

Supplementary Figure 7. **HAADF-STEM structural characterization of a non-superconducting $RuO_2(101)$ sample.** **a,** As in the superconducting $RuO_2(110)$ samples, continuous film growth is observed across the entire length of the STEM lamella. **b,** Epitaxial growth between the RuO_2 film and TiO_2 substrate is again confirmed. Here, however, the observed contrast is comparatively smooth across the film, without the clear signs of high strain observed in the $RuO_2(110)$ sample. **c,** Atomic-resolution STEM image demonstrating the high crystalline quality of the $RuO_2(101)$ sample. Inset shows the expected structure for this projection (not to scale).

ceptionally clean crystalline quality: all of the atomic columns of Ru stack uniformly in the projection of the STEM image to produce highly ordered atomic contrast. In other regions of the same film, strain gradients in the film distort the RuO_2 lattice such that the columns of Ru atoms are slightly misaligned to the electron beam projection. This local misalignment of the lattice causes the apparent blurring and more mottled contrast of the STEM image seen in Supplementary Fig. 5c.

In Supplementary Fig. 6, the same sample is studied with HAADF-STEM imaging in the orthogonal projection direction. This orientation allows us to assess the crystalline response of the RuO_2 film along the [001] in-plane direction, which is subject to a larger lattice mismatch with the TiO_2 substrate, -4.7% compressive strain. Again, Supplementary Fig. 6a confirms the continuous and epitaxial growth of the $RuO_2(110)$ thin film over the mesoscopic and macroscopic length scales relevant for interpreting the electrical transport data shown elsewhere in the manuscript. Effects of the large compressive strain along the in-plane direction of this projection are apparent in Supplementary Fig. 6a-b as characteristic V-shaped contrast in the RuO_2 film. Contributions from electron channeling in ADF-STEM imaging produces this bright/dark contrast in regions of local crystallographic strain; such contrast is a common signature of epitaxial lattice strain in many other oxide systems. Supplementary Fig. 6c shows the same structural response at atomic resolution, where—similar to Supplementary Fig. 5c—the apparent blurring of atomic columns arises from regions where the film lattice has been locally distorted.

Finally, for completeness we also performed HAADF-STEM measurements on the same non-superconducting 18.6 nm thick $RuO_2(101)$ film characterized in Fig. 1c and Fig. 2a of the main text and in Supplementary Fig. 3. Z-contrast images of this sample are shown in Supplementary Fig. 7: the film is comparably continuous and epitaxial as the superconducting $RuO_2(110)$ films we have studied, without any signatures of extended defects or secondary phase inclusions that might otherwise alter its electrical properties. In good agreement with the XRD and electrical transport data shown in Supplementary Fig. 3, the $RuO_2(101)$ film exhibits more coherent crystalline order than the more drastically strained superconducting $RuO_2(110)$ films, even over relatively large fields of view as shown in Supplementary Fig. 7b. Supplementary Fig. 7c shows that the lattice remains largely defect-free down to the atomic scale.

The data presented in Supplementary Figs. 3-6 indicate that the crystal structures of superconducting $RuO_2(110)$ films are not commensurately strained to the TiO_2 substrates. To better visualize how this partial strain relaxation manifests in real space, we employed Fourier filtering of STEM images to find edge dislocations, following the techniques described in Ref. [23]. Specifically, in Supplementary Fig. 8a (Supplementary Fig. 8c) we plot an atomic-resolution HAADF-STEM image acquired in projection along [001] (projection along [1$\bar{1}$0]) where the lateral in-plane direction is aligned with [1$\bar{1}$0] ([001], respectively). Supplementary Fig. 8b (Supplementary Fig. 8d) displays the Fourier component of this image with spatial frequencies $|q_{||}| \approx 1/d_{1\bar{1}0}$ ($|q_{||}| \approx 1/c$), which we obtained by computing the fast Fourier transform (FFT) of the image in Supplementary Fig. 8a (Supplementary Fig. 8c), and then taking the inverse FFT with the contributions of all spatial frequencies smoothly masked out except for those in a narrow region of q-space surrounding the noted $\pm q_{||}$. The horizontal dashed lines in Supplementary Fig. 8b,d represent the boundaries of the film along the out-of-plane direction.

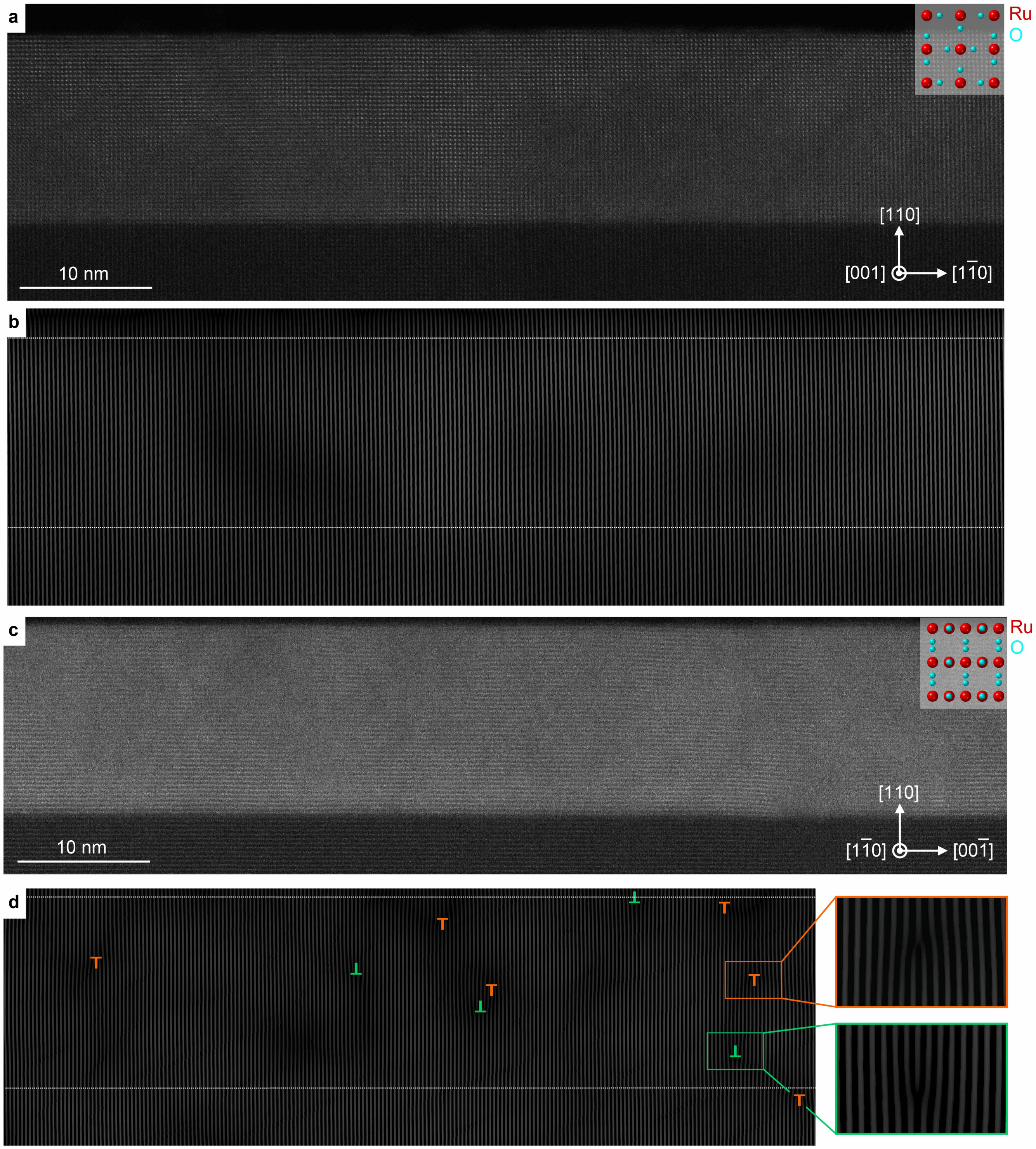

Supplementary Figure 8. **STEM imaging of edge dislocations in a superconducting $RuO_2/TiO_2(110)$ sample. a,** Atomic-resolution HAADF-STEM image in [001] projection for the same 14.2 nm thick $RuO_2(110)$ film characterized elsewhere in the manuscript by STEM, x-ray diffraction, and electrical transport. **b,** Fourier-filtered version of the image in **a**, keeping only the components having $|q_{||}| \approx 1/d_{1\bar{1}0}$ ($d_{1\bar{1}0} \approx 3.2$ Å). The horizontal dashed lines represent the boundaries of the film along the out-of-plane direction. The TiO_2 and RuO_2 lattices are continuously matched in plane over the entire 226-unit-cell-wide (73-nm-wide) field of view, without any dislocations. **c - d,** Same as in **a - b**, except in [1$\bar{1}$0] projection and isolating the Fourier component of the image having $|q_{||}| \approx 1/c$ ($c \approx 3.0$ Å). Nearly equal numbers of edge dislocations are observed in **d** with Burgers vectors of $-\mathbf{c}$ (orange) and $+\mathbf{c}$ (green), respectively, across a 204-unit-cell-wide (61-nm-wide) field of view. The insets on the right side of panel **d** are magnified by 3×.

In this representation, edge dislocations appear as topological defects in the otherwise continuous vertical streaks appearing in Supplementary Fig. 8b,d. These vertical streaks are formally the lattice points of the film and substrate crystal structures, blurred into streaks along the out-of-plane direction because we discard any high-spatial-frequency information about out-of-plane correlations of the electron density (i.e., $q_\perp \not\approx 0$) when computing the inverse FFTs. Hereafter we loosely refer to these streaks as *atomic columns*, since the contrast in these HAADF-STEM data arises predominantly from electron scattering by the atomic cores with larger Z (i.e., Ti and Ru). Dislocations indicated by green markers add one atomic column to the number of columns that exist in layers beneath it (thus relaxing tensile strain in the lateral direction), whereas dislocations indicated by orange markers remove one atomic column to the number of columns that exist in layers beneath it (thus relaxing compressive strain in the lateral direction). Therefore, a fully strain-relaxed film of $RuO_2/TiO_2(110)$ would show a collection of only green (only orange) dislocations accumulated at the substrate-film interface in Supplementary Fig. 8b (Supplementary Fig. 8d, respectively). The dislocation densities expected in this fully strain-relaxed scenario would be 1 per every $1/0.023 \approx 43$ vertical streaks for green dislocations in Supplementary Fig. 8b, and 1 per every $1/0.047 \approx 21$ vertical streaks for orange dislocations in Supplementary Fig. 8d.

In marked contrast to this behavior, zero dislocations are observed across a 226-unit-cell-wide field of view in Supplementary Fig. 8b. Furthermore, although a significantly higher density of dislocations is present across the 204-unit-cell-wide field of view in Supplementary Fig. 8d, there are nearly equal numbers of edge dislocations having Burgers vectors of $-\mathbf{c}$ (orange) and $+\mathbf{c}$ (green), respectively, and the dislocations are rather uniformly distributed throughout the entire thickness of the film. These observations imply that throughout a sizable volume fraction of the superconducting film, the crystal structure is, on average, much closer to the commensurately-strained limit than to the fully-relaxed limit. We note that this agrees well with the distribution of x-ray scattering intensities plotted in the RSMs for this sample and others in Supplementary Figs. 9-10. Based on these data, we suggest that it is appropriate to consider the local strain gradients that inevitably accompany the nucleation of dislocations in $RuO_2(110)$ to be sample-dependent perturbations to significantly larger average components of the substrate-imposed strain fields that are present throughout all films shown in the manuscript. Because superconductivity is an essentially mean-field phenomenon, we believe that the latter average components of the strain fields in $RuO_2(110)$ are the key ingredients for stabilizing superconductivity with transition temperatures at least an order of magnitude larger than in bulk RuO_2; finer details of the local strain gradients probably determine finer details of the superconductivity, such as the exact sample-dependent T_cs measured by non-bulk-sensitive probes of superconductivity, such as resistivity. Since our platform for applying strain enables scanning-probe measurements of the superconducting condensate, future experiments may be able to provide direct experimental evidence to support these general expectations of mesoscale or nanoscale strain inhomogeneity resulting in spatially inhomogeneous superconductivity [24].

Supplementary Note 4: STRUCTURAL AND ELECTRICAL CHARACTERIZATION DATA FOR $RuO_2(110)$ FILMS OF DIFFERENT THICKNESSES

Figure 2b-c of the main text shows that the superconducting T_cs of RuO_2 thin films synthesized on $TiO_2(110)$ substrates depend sensitively on the thickness of the films, t. We do not purport to completely understand this empirical observation, but Supplementary Figs. 9-10 include several additional pieces of structural and electrical characterization data for this same thickness series of $RuO_2(110)$ samples that constrain potential explanations.

In Supplementary Fig. 9a-b, we plot x-ray reflectivity (XRR) data taken at low incident angles, and XRD data taken near the 110 Bragg peaks of the film and substrate, both acquired along the specular CTRs using Cu-Kα radiation. Finite-thickness fringes are present over a wide range of angles in both data sets, evidencing (from reflectivity) atomically abrupt interfaces of the films with the substrates and with vacuum, and (from diffraction) comparable levels of crystallinity along the out-of-plane direction across samples. Furthermore, the spacings between secondary maxima on either side of the primary film Bragg peaks in XRD match the spacings between the low-angle XRR fringes, suggesting that the crystal structures of all films are essentially homogeneous along the out-of-plane direction. The film thicknesses t listed in Fig. 2b-c of the main text and in Supplementary Figs. 9-10 are obtained by directly fitting the XRR data in Supplementary Fig. 9a using a genetic algorithm, which yields sub-nanometer roughnesses in all cases in the refined models.

Given that there are no obvious differences in film morphology or out-of-plane crystallinity between $RuO_2(110)$ samples with different t, an alternative explanation that may account for the thickness-dependent superconducting T_cs is the proliferation of misfit dislocations in thicker films that progressively relax the epitaxial—i.e., in-plane—strains; in this scenario, it may be that partially strain-relaxed $RuO_2(110)$ films have higher (average) superconducting T_cs compared with fully commensurately strained $RuO_2(110)$ films. To investigate this possibility, in Supplementary Fig. 9c-e we plot line cuts of the intensity versus $q_{||}$ extracted from RSMs near the 220, 310, and 332 Bragg reflections of the $RuO_2(110)$ thin films and TiO_2 substrates. The raw data for all

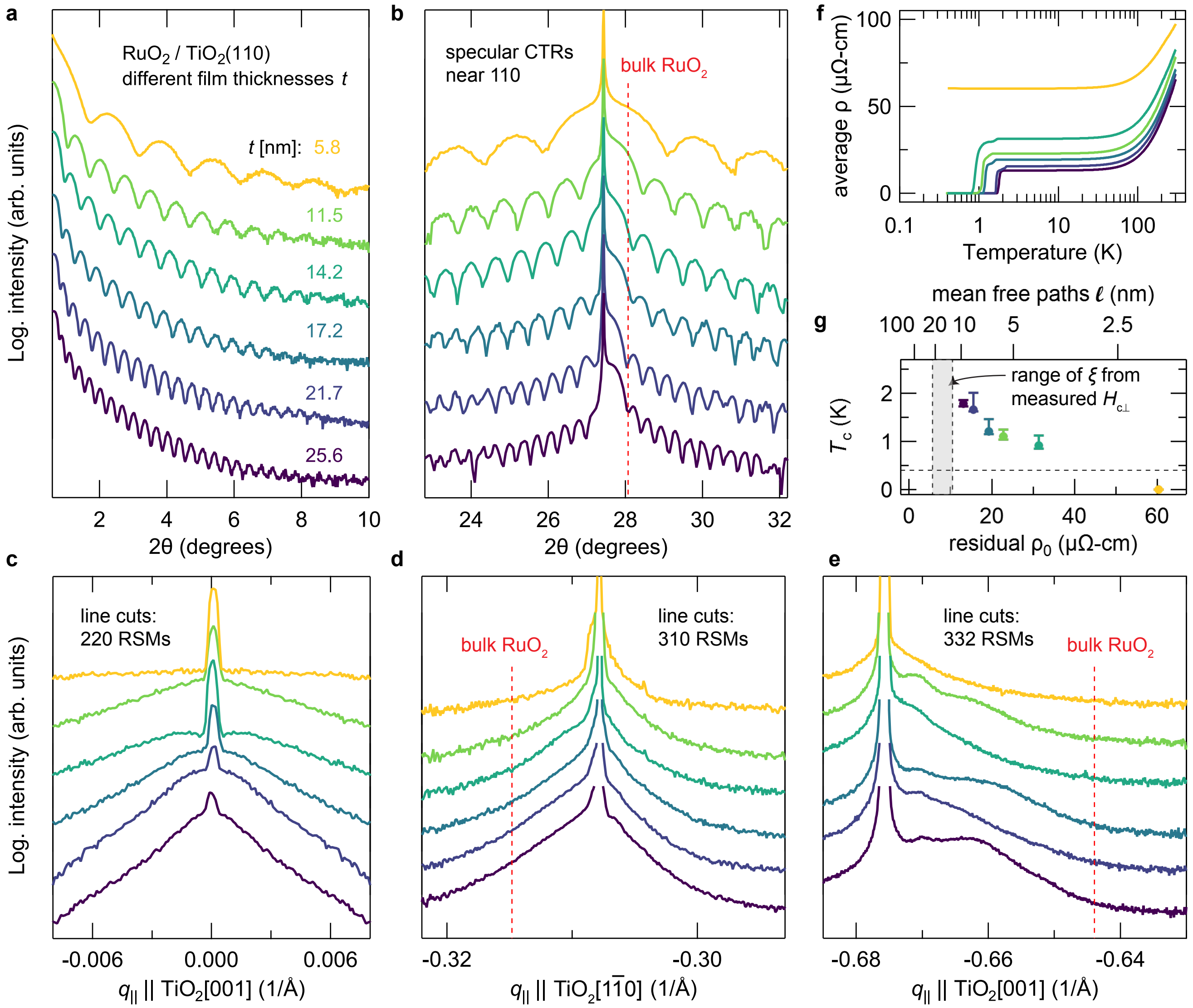

Supplementary Figure 9. **Evolution of crystal structure and electrical transport behavior as a function of film thickness for $RuO_2/TiO_2(110)$. a,** X-ray reflectivity and **b,** x-ray diffraction data along the specular CTR show comparable levels of flatness and crystalline order along the out-of-plane direction for all samples. **c - e,** Average line cuts versus $q_{||}$ through the 220, 310, and 332 RSMs (fully **q**-resolved data are shown in Supplementary Fig. 10) indicate that all samples with $t > 5.8$ nm exhibit partial strain relaxation. **f - g,** Zero-field $\rho(T)$ data show that thinner films generally have higher residual resistivities ρ_0 and lower superconducting T_cs. T_cs in **g** are taken as the temperatures at which $\rho(T)$ crosses 50% of the normal-state ρ_0, and error bars indicate the temperatures at which $\rho(T)$ crosses 90% and 10% of ρ_0, respectively. The horizontal dashed line in **g** represents the base temperature attainable in our cryostat (0.4 K), and the gray-shaded region indicates the range of superconducting coherence lengths ($\xi = 12 - 22$ nm) extracted from magnetoresistance measurements of the upper critical fields for ten different $RuO_2(110)$ thin films. Comparisons of these ξ with the mean free paths ℓ (top axis of **g**) that correspond to the measured ρ_0 (bottom axis of **g**) indicates that superconductivity persists in the dirty limit $\ell < \xi$.

RSMs are plotted in Supplementary Fig. 10 using logarithmic false color scales; the line cuts in Supplementary Fig. 9c-e are averaged over the ranges of $q_\perp$ between the dashed white lines in Supplementary Fig. 10. All of the samples except the thinnest film exhibit diffuse scattering surrounding the CTRs, indicating that partial strain relaxation onsets between film thicknesses of 5.8 nm and 11.5 nm for the growth conditions used in this work to synthesize $RuO_2/TiO_2(110)$ samples. Since the in-plane lattice mismatches between RuO_2 and TiO_2 are highly anisotropic for the (110) orientation, it might also be expected that the substrate-imposed compressive strain along [001] (-4.7%) starts to relax at smaller film thicknesses than the tensile strain along $[1\bar{1}0]$ ($+2.3\%$) [17, 19]. The off-specular RSMs in Supplementary Fig. 10b-c qualitatively agree with this expectation, inasmuch as finite-

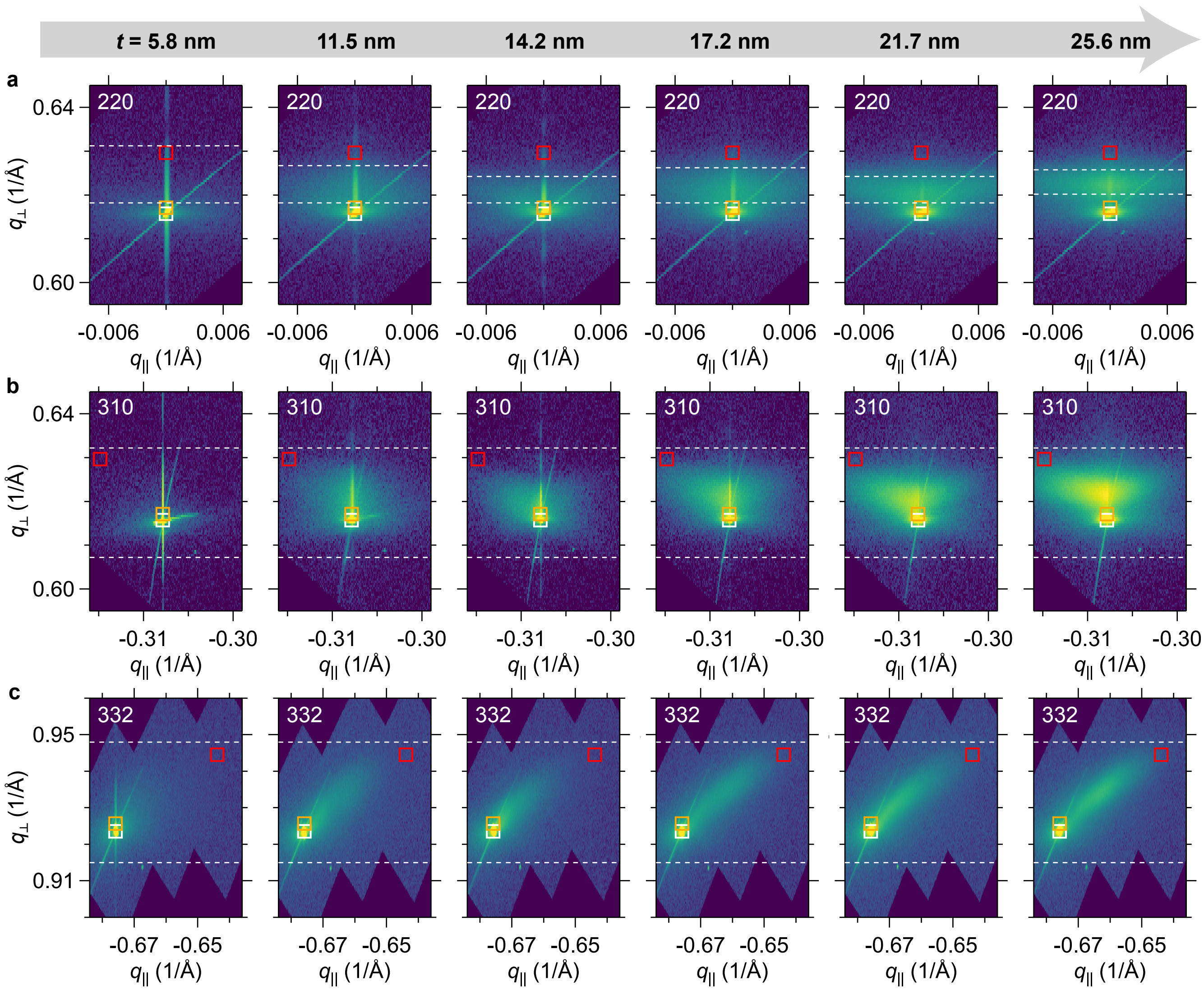

Supplementary Figure 10. **RSMs for $RuO_2/TiO_2(110)$ samples with different film thicknesses, t. a,** RSMs along the specular CTR near the 220 Bragg reflections for films with increasing t, moving from left to right. $q_{||}$ is aligned with [001] in all panels, although the phenomenology is similar with $q_{||}$ along $[1\bar{1}0]$, cf. Supplementary Fig. 4. **b,** Thickness-dependent RSMs near the off-specular 310 Bragg reflections where $q_{||}$ is purely along $[1\bar{1}0]$. **c,** Same as **b**, but near the 332 Bragg reflections where $q_{||}$ is purely along [001]. White, red, and orange squares represent the central peak positions expected for bulk TiO_2, bulk RuO_2, and commensurately strained $RuO_2(110)$, as in Supplementary Fig. 3. The line cuts plotted in Supplementary Fig. 9c-e are averaged over the ranges of $q_\perp$ of the RSMs between the horizontal white dashed lines.

thickness fringes can still be observed along the CTRs in the RSMs near 310 for films up to at least $t = 17.2$ nm, whereas only the $t = 5.8$ nm film shows a contribution to the coherent CTR scattering in the RSMs near 332 that clearly rises above the contributions of the substrate.

Although signatures of scattering from partially strain-relaxed $RuO_2(110)$ are manifestly present in the data for all of the superconducting samples in Supplementary Fig. 9—namely, broader distributions of intensity versus $q_{||}$ that asymmetrically gain weight towards the positions expected for bulk RuO_2 as the film thickness increases—it remains somewhat ambiguous whether this data can be interpreted in a straightforward manner to gain insight into what levels of strain optimize the superconducting T_cs in RuO_2. Strain relaxation in oxide thin films often occurs inhomogeneously, with a mixture of commensurately strained and partially relaxed material [25]. Indeed, examining the transport data for these same samples in Fig. 2b of the main text and in Supplementary Fig. 9f, it is tempting to ascribe the multi-stage behavior of the superconducting transitions to temperature-dependent Josephson coupling of regions of the films under different amounts of strain with correspondingly different "local" T_cs; similar behavior has been described

theoretically [26] and observed experimentally in patterned niobium islands on gold substrates [27]. However, because of the close proximity of the substrate Bragg peaks along $q_\perp$ ($d_{110} = 3.248$ Å) with the positions expected for commensurately strained $RuO_2(110)$ ($d_{110} = 3.241$ Å), it is difficult to disentangle their respective contributions to the total scattering observed in XRD.

Despite these complications in quantitatively analyzing the XRD results, we can use the values of t obtained from XRR to plot the normalized resistance versus temperature curves from Fig. 2b of the main text in terms of absolute resistivities, as shown in Supplementary Fig. 9f. From these data, a robust correlation between the superconducting T_cs and the residual resistivities ρ_0 immediately becomes apparent, as displayed in Supplementary Fig. 9g. As noted in the main text, this correlation may suggest that the primary effect of reducing t is to enhance the relative importance of elastic scattering off disorder near the substrate-film interfaces, which is known to decrease T_c in numerous families of thin-film superconductors, both conventional [28] and unconventional [29]. It is largely outside the scope of this paper to contribute meaningful data to ongoing research efforts investigating the mechanism underlying thickness-induced suppressions of T_c that are ubiquitously observed for superconducting films in the two-dimensional limit [30]; however, in passing we note that the residual resistivity ($\rho_0 = 60\,\mu\Omega$-cm) of the thinnest ($t = 5.8$ nm) non-superconducting ($T_c < 0.4$ K) $RuO_2(110)$ film shown in Supplementary Fig. 9 corresponds to a sheet resistance of $R_s = \rho_0/t = 0.10$ k$\Omega/\square$. This value of R_s is about 60 times less than the Cooper pair quantum of resistance $h/(2e)^2 = 6.45$ k$\Omega/\square$ that was empirically noted to separate insulating from superconducting ground states in ultrathin films of numerous elemental metals [31, 32]; this indicates that quantitatively different physics is likely operative here in suppressing T_c, which may place ultrathin films of $RuO_2(110)$ in closer proximity to the *anomalous metal* regime that was shown to occur at weaker levels of disorder in Ref. [33].

We believe that identifying the exact mechanism underlying the strain-stabilized superconductivity in $RuO_2(110)$ is also well beyond the scope of the current paper. Phase-sensitive measurements of the superconducting order parameter—and/or momentum-resolved measurements of the superconducting gap magnitude—are notoriously challenging in multi-band materials with small (sub-meV) gaps, and a definitive answer to whether the pairing is (un)conventional will have to wait until such data become available. With that qualification in mind, it is natural to consider whether the T_c versus ρ_0 behavior displayed in Supplementary Fig. 9g can shed any light on the answer to this question. To address this possibility, we need to convert measured properties of the normal-metal and superconducting states into comparable characteristic length scales. In unconventional low-temperature superconductors with sign-changing order parameters, such as Sr_2RuO_4 [34], superconductivity is completely suppressed by non-magnetic impurity scattering whenever the normal-state mean free path ℓ is comparable with the clean-limit superconducting coherence length ξ_0—i.e., $T_c \to 0$ K if $\ell \approx \xi_0$. In conventional superconductors, by contrast, superconductivity persists even in the dirty limit $\ell << \xi_0$.

On the top horizontal axis of Supplementary Fig. 9g, we indicate approximate values of ℓ corresponding to the measured values of ρ_0 on the bottom horizontal axis; these numbers are computed following the analysis of Glassford et al. [3], who used the DFT-computed plasma frequencies and Fermi velocities for bulk RuO_2 to obtain $\ell\,[\text{nm}] = 3.6\cdot 35/\rho\,[\mu\Omega\text{-cm}]$. Because of various uncertainties implicit in these estimations, we neglect any strain-dependent changes in Fermiology that will, of course, quantitatively renormalize the precise relationship between ℓ and ρ for $RuO_2(110)$. To compare with ℓ, we also include a gray-shaded region on Supplementary Fig. 9g corresponding to the range of average in-plane superconducting coherence lengths ξ we extracted experimentally from magnetoresistance measurements of the perpendicular upper critical magnetic fields $H_{c\perp}$ for ten different superconducting $RuO_2(110)$ samples, following the procedures detailed in Supplementary Note 2. As noted previously, these values of ξ likely represent a lower bound for what the clean-limit ξ_0 would be in the absence of extrinsic defects in the films that impede vortex motion. In any case, we find that superconductivity robustly persists in $RuO_2(110)$ even when $\ell < \xi < \xi_0$; for example, the samples shown here with $T_c = 0.9 - 1.8$ K have measured residual resistivities corresponding to mean free paths $\ell = 4.0 - 9.6$ nm, which are all less than the range of measured superconducting coherence lengths, $\xi = 12 - 22$ nm. Therefore, whatever the superconducting pairing mechanism is in $RuO_2(110)$, these empirical considerations demonstrate that it is rather insensitive to defect scattering.

Conceptually, perhaps the most straightforward test of our proposal that substrate-imposed strains are an essential ingredient in stabilizing superconductivity in $RuO_2(110)$ would be to perform electrical transport measurements for a thick $RuO_2(110)$ film where the epitaxial strains are almost completely relaxed. In reality, however, such efforts are complicated by the observation that the $+2.3\%$ tensile strain along $[1\bar{1}0]$ is not released in sufficiently thick films of $RuO_2(110)$ by the nucleation of misfit dislocations; instead, cracks form in such samples (in our studies, for $t \gtrsim 30$ nm) that propagate through the entire thickness of the film and even tens of nanometers into the substrate.

In Supplementary Fig. 11a-b, we show HAADF-STEM images of such oriented micro-cracks for the same 48 nm thick $RuO_2(110)$ film characterized by ARPES and LEED in Fig. 4 of the main text and in Supplementary Figs. 14-15. Although these images evidence strong interfacial bonding between film and substrate—which is certainly crucial to maintain high levels of strain throughout

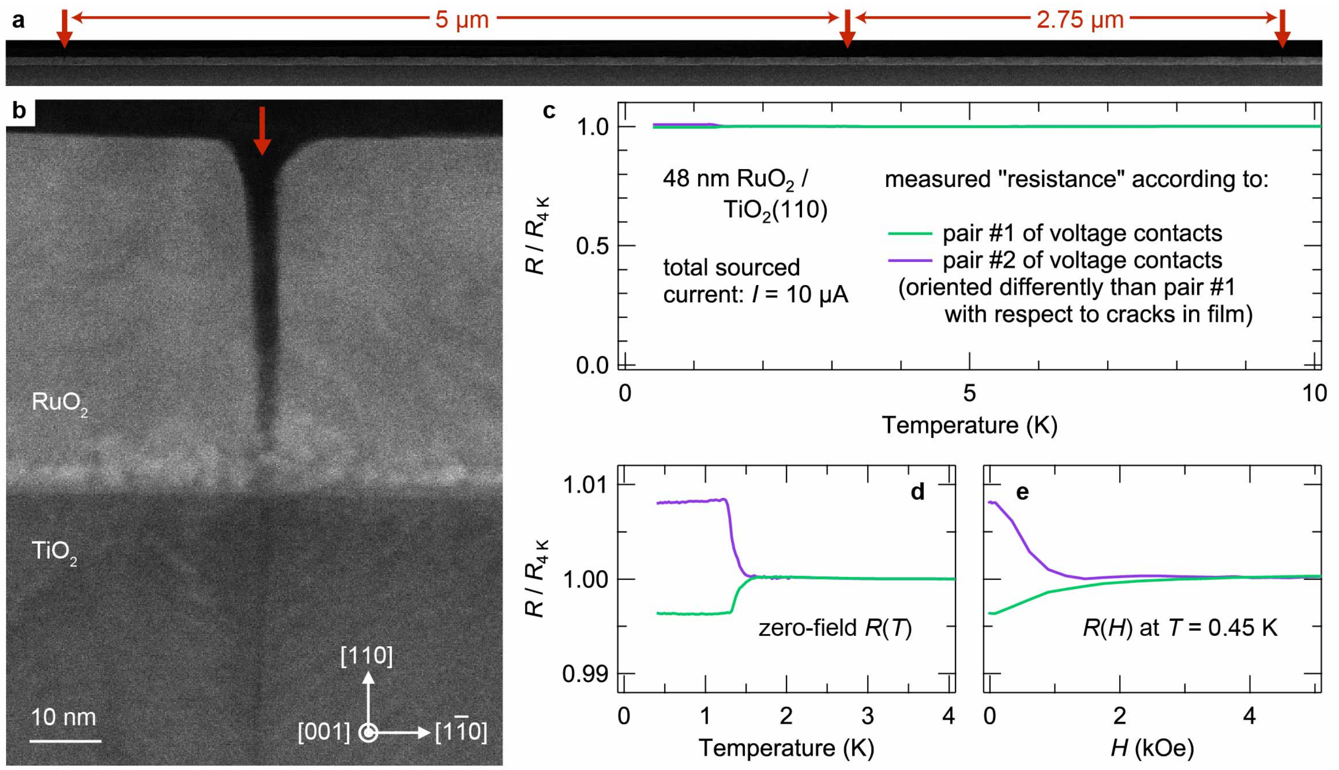

Supplementary Figure 11. **Structural and electrical characterization of a cracked 48 nm thick $RuO_2/TiO_2(110)$ sample.** **a,** HAADF-STEM image of the sample over a wide field of view, showing an anisotropically oriented network of cracks (red arrows) each separated by a few microns. Note that the cracks appear only in projection view along [001]—i.e., perpendicular to [1$\bar{1}$0], which is the in-plane direction of the film under tensile strain. **b,** Magnified image of a crack from **a**. **c,** Zero-field "resistance" versus temperature data for two different pairs of voltage contacts oriented differently with respect to the network of cracks visualized in **a** and **b**. **d,** Same $R(T)$ data as in **c**, plotted on a greatly expanded vertical scale. **e,** "Resistance" versus externally applied magnetic field data for the pairs of voltage contacts from **c** and **d**, taken at fixed temperature $T = 0.45$ K.

the thinner $RuO_2(110)$ films we characterize elsewhere in the manuscript—the strongly anisotropic nature of the cracks makes the distribution of current flow through such samples extremely non-uniform. Accordingly, putative measurements of electrical "resistance" $R(T, H)$ displayed in Supplementary Fig. 11c-e should be more pedantically interpreted as the voltage difference measured between two voltage contacts, divided by the total current sourced through two other contacts placed elsewhere on the film. Unsurprisingly, the results thus obtained for R depend in an essential way on the orientation of the voltage contacts relative to the cracks in the film: in Supplementary Fig. 11c-d we observe either a downturn (green trace) or upturn (purple trace) in the apparent R as the temperature is decreased below $T_c = 1.3 - 1.5$ K, followed by plateaus at lower temperatures. In Supplementary Fig. 11e, we show that these temperature-induced anomalies in R can be suppressed in both cases by applying small magnetic fields $H_{c\perp} < 3$ kOe at fixed $T = 0.45$ K, confirming that they result from (inhomogeneous) patches of superconductivity. Irrespective of whether the measured R decreases or increases at (T_c, H_c), we emphasize that the fractional changes in resistance induced by superconductivity in this 48 nm thick $RuO_2(110)$ film are less than 1% of the residual normal-state resistances $R_{4\ \mathrm{K}}$, in marked contrast to the full 100% drops to zero resistance exhibited by all thinner, more highly strained, uncracked $RuO_2(110)$ films shown in other figures.

Supplementary Note 5: DENSITY FUNCTIONAL THEORY CALCULATIONS

Structural relaxations

One of the central themes of this work is the exploration of strain-induced changes to the electronic structure in epitaxial thin films of RuO_2 subject to biaxial epitaxial strains imposed by differently oriented rutile TiO_2 substrates. To model this situation computationally within the framework of density functional theory (DFT), we started by using the Vienna Ab Initio Software Package [35, 36] to perform full structural

relaxations (of lattice parameters and internal coordinates) to minimize the DFT + U-computed total energy of RuO_2 in the ideal tetragonal rutile crystal structure (space group #136, $P4_2/mnm$). Structural relaxations employed the same exchange-correlation functional and calculational parameters as for the DFT + U ($U = 2$ eV) calculations described in the Methods section of the main text, and forces were converged to < 1 meV/Å.

Throughout the main text and supplementary information, we refer to DFT results for this minimum energy structure as "bulk RuO_2". The actual lattice parameters for this structure, ($a_{\rm bulk} = 4.517$ Å, $c_{\rm bulk} = 3.130$ Å), overestimate the experimentally measured lattice parameters at 295 K for RuO_2 single crystals of ($a = 4.492$ Å, $c = 3.106$ Å) by $< 1\%$, due to well-established deficiencies of the generalized gradient approximation. With the former as the bulk reference structure, we then simulated biaxial epitaxial strains to (110)-oriented TiO_2 substrates by performing constrained structural relaxations for RuO_2 in which the in-plane lattice parameters $c = (1 - 0.047) \times c_{\rm bulk}$ and $d_{1\bar{1}0} = (1 + 0.023) \times d_{1\bar{1}0,\ \rm bulk}$ were held fixed, while the out-of-plane lattice constant d_{110} and all other internal coordinates of the structure were allowed to relax so as to minimize the total energy. The fixed compression and expansion of c and $d_{1\bar{1}0}$, respectively, correspond to the experimentally measured lattice mismatches between TiO_2 and RuO_2 single crystals at 295 K [13, 14].

Within this scheme, DFT + U predicts that commensurately strained RuO_2(110) thin films will have an out-of-plane lattice constant $d_{110} = (1 + 0.017) \times d_{110,\ \rm bulk}$, which compares reasonably well with the 2.0% expansion of d_{110} measured experimentally on a 5.8 nm thick RuO_2(110) film. Because the splitting of d_{110} and $d_{1\bar{1}0}$ in strained RuO_2(110) breaks the non-symmorphic glide plane symmetry of the parent rutile structure, we used a base-centered orthorhombic structure (space group #65, $Cmmm$) with lattice constants of $c \times 2d_{1\bar{1}0} \times 2d_{110}$ for DFT simulations of RuO_2(110). The primitive unit cell of this $Cmmm$ structure contains the same number of atoms as the parent rutile unit cell, so there is no apparent doubling and/or folding of the bands in spaghetti plots that compare the bandstructures of RuO_2(110) and bulk RuO_2, such as in Supplementary Fig. 12 or Fig. 4a of the main text.

To simulate the electronic structure of commensurately strained (101)-oriented RuO_2 thin films, we adopted a slightly different approach, since it is not straightforward to perform constrained structural relaxations with DFT in such a low-symmetry situation. Specifically, we took the rutile b axis to be under +2.3% tension, i.e. $b = (1 + 0.023) \times b_{\rm bulk}$, as dictated by the lattice mismatch of RuO_2 with TiO_2 along this direction (cf. Supplementary Fig. 3d). On the other hand, the lengths of the rutile a and c axes are free to adjust their lengths, but are subject to the simultaneous constraints:

$$\sqrt{a^2 + c^2} = \sqrt{\left(a_{\rm TiO2}\right)^2 + \left(c_{\rm TiO2}\right)^2} = 5.464 \text{ Å} \quad (3)$$

$$|\mathbf{q}_{202}| = \sqrt{\left(\frac{2}{a}\right)^2 + \left(\frac{2}{c}\right)^2} = 0.792 \text{ Å}^{-1} \quad (4)$$

$$|\mathbf{q}_{103}| = \sqrt{\left(\frac{1}{a}\right)^2 + \left(\frac{3}{c}\right)^2} = 0.998 \text{ Å}^{-1} \quad (5)$$

$$|\mathbf{q}_{402}| = \sqrt{\left(\frac{4}{a}\right)^2 + \left(\frac{2}{c}\right)^2} = 1.114 \text{ Å}^{-1}. \quad (6)$$

Supplementary Eq. (3) ensures that the film is lattice matched to the TiO_2 substrate along the $[\bar{1}01]$ in-plane direction (cf. Supplementary Fig. 3c), and Supplementary Eqs. (4)-(6) ensure that the d-spacings for the HKL = 202, 103, and 402 Bragg reflections reproduce the values we measured experimentally for a commensurately strained RuO_2(101) film in Fig. 2a of the main text, in Supplementary Fig. 3c, and in analogous RSM data taken around 402 (a proper, i.e. highly overconstrained, lattice constant refinement would of course include data for many more reflections). Note that in deriving these equations, we assumed for simplicity that the angle between the rutile a and c axes remains 90° in epitaxially strained films; small deviations away from this limit should be expected in reality, since this orthogonality is not guaranteed by any symmetry or constraint of the system. Nonetheless, finding the best-fit solution to Supplementary Eqs. (3)-(6) gives lattice constants of ($a = 4.501$ Å, $c = 3.077$ Å) in absolute units; dividing through by the experimentally measured lattice constants of bulk RuO_2 yields $a = (1 + 0.002) \times a_{\rm bulk}$ and $c = (1 - 0.009) \times c_{\rm bulk}$ as appropriately scaled inputs for DFT simulations. With $a \neq b \neq c$ and all angles between the primitive unit cell translations equal to 90°, the crystal structure for RuO_2(101) DFT simulations was taken as the primitive orthorhombic space group #58, $Pnnm$. Supplementary Table 1 summarizes all parameters of the crystal structures used in DFT simulations for bulk RuO_2, RuO_2(110), and RuO_2(101).

Effects of adding $+U$

In Supplementary Fig. 12, we show the effects of including an ad hoc static mean-field $+U$ term on the Ru sites in DFT calculations. Adding such a phenomenological term to the Kohn-Sham Hamiltonian shifts the bands relative to each other (up/down in energy) so as to force the orbital occupancies towards integer fillings, rather than also shrinking the bandwidths of the quasiparticle excitations, as would occur in a more realistic

Supplementary Table I. Crystal structures used in DFT simulations (all units of length are given in Å)

Name	Space group	a_{rutile}	b_{rutile}	c_{rutile}	$d_{1\bar{1}0}$	d_{110}	Ru - apical O bond length(s)	Ru - equatorial O bond length(s)
bulk RuO_2 - expt.	#136	4.492	4.492	3.106	3.176	3.176	1.941	1.984
bulk RuO_2 - DFT	#136	4.517	4.517	3.130	3.194	3.194	1.945	2.002
RuO_2(110) - DFT	#65	4.606	4.606	2.982	3.266	3.249	1.946, 1.957	1.980, 1.984
RuO_2(101) - DFT	#58	4.525	4.618	3.101	3.233	3.233	1.969	2.000

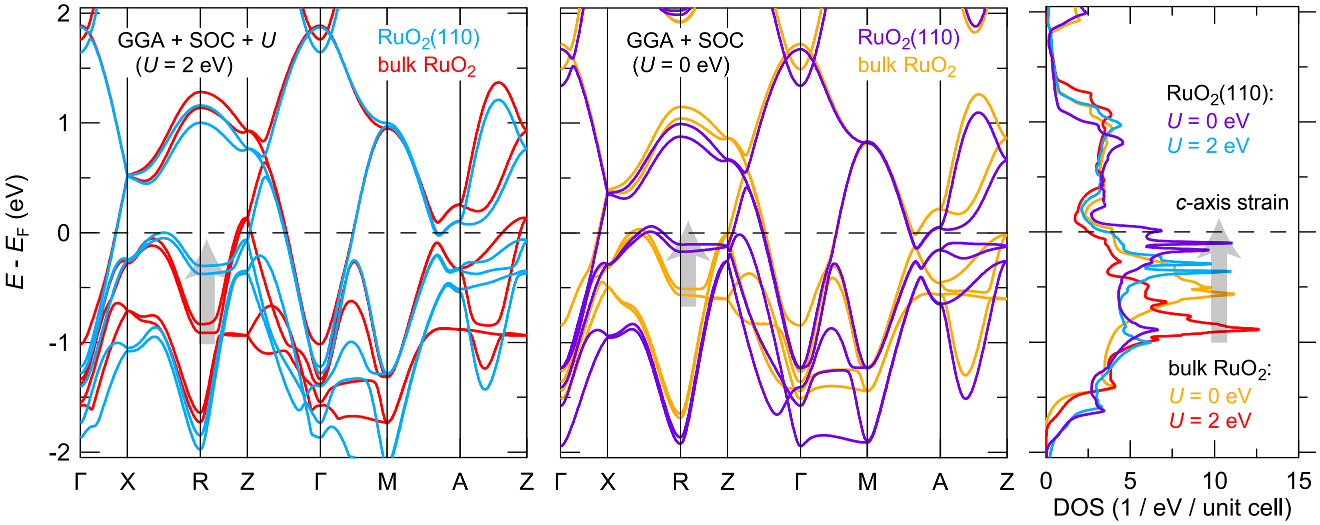

Supplementary Figure 12. **Strain dependence of the electronic structure of RuO_2, according to DFT (+ U).**

theory that includes dynamical electron-electron interactions. The blue and red traces are reproduced from the non-magnetic DFT + U ($U = 2$ eV) results presented in Fig. 4a of the main text; the purple and orange traces are the results of repeating GGA + SOC calculations for the same RuO_2(110) and bulk RuO_2 crystal structures, respectively, but now setting $U = 0$. Irrespective of U, both sets of calculations show a shift of the $d_{||}$-derived flat bands towards E_F and concomitant enhancement of the density of states (DOS) near E_F when the amount of c-axis compression is increased upon going from bulk RuO_2 to RuO_2(110), as indicated by the gray arrows. While these strain-dependent trends in the electronic structure are robust against fine-tuning of parameters employed in the calculations, Supplementary Fig. 12 also suggests that the calculated positions of the peaks in the DOS and the exact values of the DOS near E_F should not be taken too seriously, as there are considerable theoretical uncertainties in these quantities, depending on the choice of U. We leave a complete treatment of the effects on the electronic structure of commensurate $\mathbf{Q}_{\mathrm{AFM}} = (1\,0\,0)$ antiferromagnetic spin-density wave order [13, 37] to future studies [38], because it is not possible in standard DFT-based approaches to stabilize self-consistent solutions for the spin densities that have small values of the ordered magnetic moment comparable to those measured experimentally ($\approx 0.05\,\mu_B$/Ru) [13].

Supplementary Note 6: DETERMINATION OF OUT-OF-PLANE MOMENTA PROBED BY ANGLE RESOLVED PHOTOEMISSION SPECTROSCOPY

Figure 3d-f of the main text compares the electronic structure of a 7 nm thick RuO_2/TiO_2(110) sample experimentally measured by angle-resolved photoemission spectroscopy (ARPES) with the results of DFT + U simulations. To make this comparison, it is necessary to determine what range of out-of-plane momenta $k_z = k_{110}$ in the initial state are probed by ARPES at a given final-state kinetic energy and momentum. This was established by plotting the DFT-computed $E(\mathbf{k})$ dispersions on top of the experimentally measured spectra along several one-dimensional cuts through momentum space measured over a small range of kinetic energies corresponding to near-E_F states at the given photon energy (21.2 eV), and allowing k_z to vary in the calculations so as to best match the experimental data.

Supplementary Fig. 13 shows representative examples of this procedure for experimental spectra taken along

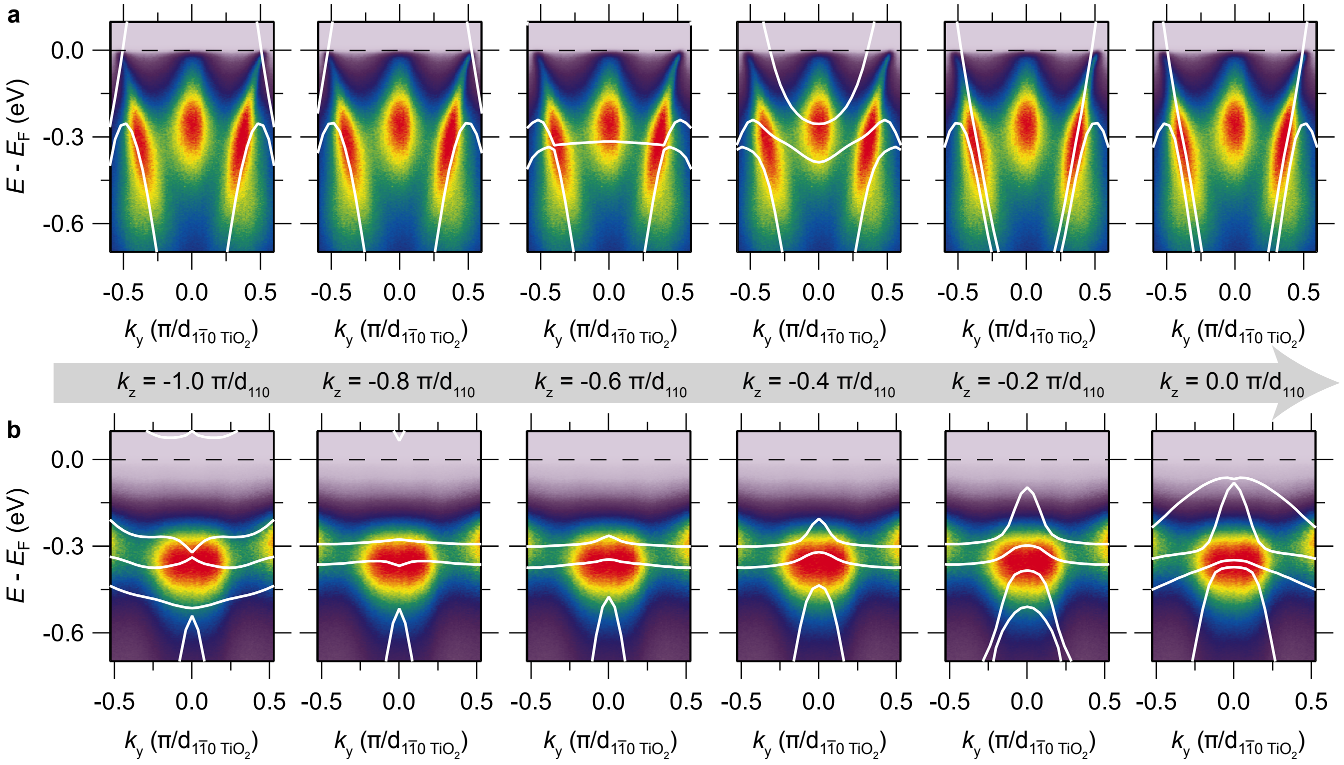

Supplementary Figure 13. **Determination of k_z probed by ARPES for RuO_2(110).** Experimental ARPES spectra for a 7 nm thick RuO_2/TiO_2(110) sample reproduced from **a,** Fig. 3f and **b,** Fig. 3e of the main text, overlaid with the band dispersions from DFT + U ($U = 2$ eV) calculations in white. For the data in **a,** $k_x = k_{001}$ is fixed at zero and in **b,** $k_x = \pi/c$. Moving from left to right, k_z is incremented in the DFT simulations in steps of 0.2 π/d_{110} starting from -1.0 π/d_{110}.

the one-dimensional cuts shown in Fig. 3f (top row, $k_x = k_{001}$ fixed at zero) and in Fig. 3e (bottom row, $k_x = \pi/c$) of the main text. Moving from left to right, k_z is incremented in the DFT simulations in steps of 0.2 π/d_{110} starting from -1.0 π/d_{110}. For the panels in Supplementary Fig. 13a, the k_Fs and electron-like character of the band crossing E_F are best fitted by calculations with k_z in the range $-0.2 \rightarrow 0.0\ \pi/d_{110}$. Likewise, for the panels in Supplementary Fig. 13b, k_z values in the range $-0.6 \rightarrow -0.3\ \pi/d_{110}$ best reproduce the measured spectra, although the results here are more ambiguous because of the insensitivity of the flat-band energies to the precise value of k_{110}. Therefore, we took the range of reduced initial-state out-of-plane momenta probed at normal emission ($k_x = k_y = 0$) to be $k_{z,i} = -0.1 \pm 0.1\ \pi/d_{110}$. Assuming a free-electron-like model of final states, the final-state $k_{z,f}$ is given by the expression

$$k_{z,f} = \sqrt{\frac{2m_e(E_k\cos^2\theta + V_0)}{\hbar^2}} = \frac{2\pi}{2d_{110}}N + k_{z,i}\ , \quad (7)$$

where m_e is the free-electron mass, E_k is the kinetic energy of the photoelectrons, θ is the emission angle relative to the surface normal, V_0 is the inner potential, and $2d_{110}$ is the spacing between equivalent lattice points along the out-of-plane direction (N can adopt any integer value). Substituting $E_k = 16.6 \pm 0.3$ eV, $\theta = 0°$, $k_{z,i} = -0.1 \pm 0.1\ \pi/d_{110}$, and $d_{110} = 3.23$ Å into Supplementary Eq. (7), we find that an inner potential of 13.7 ± 2.3 eV is compatible with our determination of $k_{z,i}$. Taking this same value of V_0 and setting $\theta = 30{-}35°$ in Supplementary Eq. (7)—as is appropriate for the experimental data in the panels displayed in Supplementary Fig. 13b—yields $k_{z,i} = -0.35 \pm 0.17\ \pi/d_{110}$; visual inspection of the DFT bands for this range of $k_{z,i}$ show that the calculations also reproduce the experimental spectrum reasonably well in this region of the Brillouin zone. The curved green planes drawn in the Brillouin zone schematic in Fig. 3c of the main text are constructed by evaluating Supplementary Eq. (7) with $V_0 = 13.7$ eV and $N = 3$ for all (k_x, k_y), and accounting for an intrinsic uncertainty of $\approx 0.2\ \pi/d_{110}$ in k_z owing to the finite elastic escape depth of photoelectrons, which we take to be ≈ 5 Å.

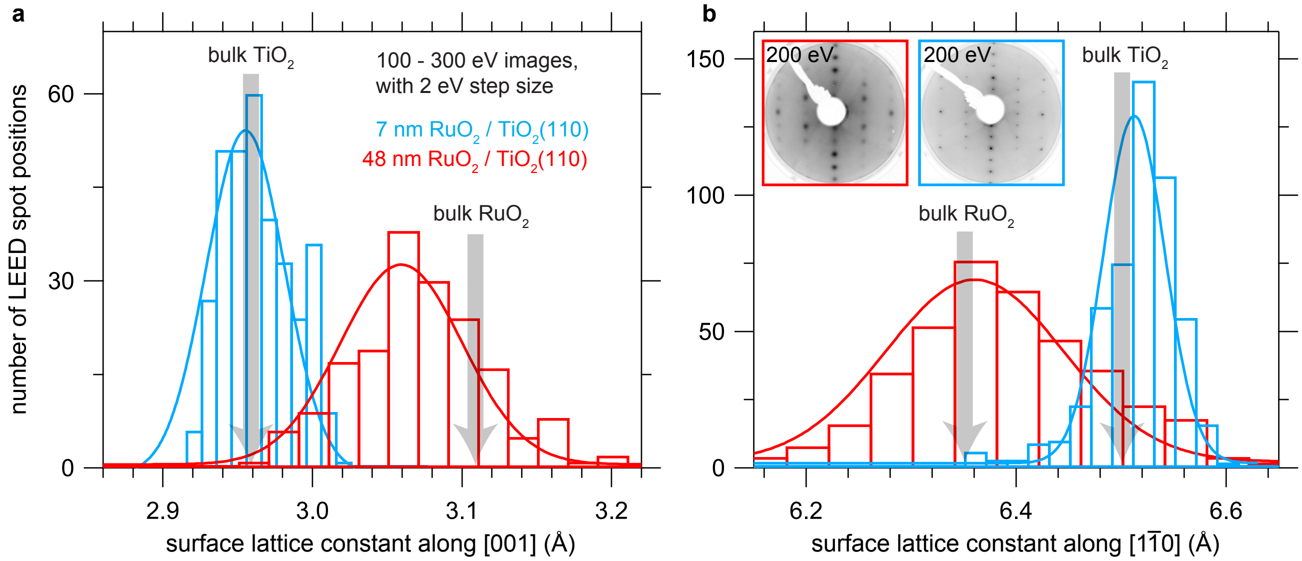

Supplementary Figure 14. **Surface lattice constant refinement by LEED spot position analysis.** Results are shown for two $RuO_2(110)$ films of different thicknesses, 7 nm (blue) and 48 nm (red). The histograms in panels **a** and **b** summarize the measured values for c and $2d_{1\bar{1}0}$, respectively. For reference, the gray arrows indicate the lattice parameters expected for a commensurately strained film (bulk TiO_2) and a fully strain-relaxed film (bulk RuO_2).

Supplementary Note 7: SURFACE LATTICE CONSTANT REFINEMENT

In Supplementary Fig. 14, we present the results of a surface lattice constant refinement for two different $RuO_2/TiO_2(110)$ films of different thicknesses, 7 nm and 48 nm. For both samples we acquired many low-energy electron diffraction (LEED) images at normal incidence using incident energies ranging from $E = 100 - 300$ eV in 2 eV steps; for examples of the raw data, the insets in Supplementary Fig. 14b contain representative images taken at 200 eV. For each image, we located the positions of all visible spots and indexed the spots according to their in-plane momentum transfer values $\mathbf{q}_{||} = 2\pi(H/c, K/2d_{1\bar{1}0})$, where H and K are integers and, by our convention, H defines the magnitude of $\mathbf{q}_{||}$ along [001] (nearly horizontal in the images in Supplementary Fig. 14), and K defines the magnitude of $\mathbf{q}_{||}$ along [1$\bar{1}$0] (nearly vertical). We then calculated the distance of all spots from the specular $\mathbf{q}_{||} = (0, 0)$ reflection and converted these image distances D (in pixel space) to scattering angles $\sin(\theta)$ (where θ is the angle of each diffracted electron beam relative to the surface normal) based on $D \rightarrow \sin(\theta)$ calibrations that were independently determined from reference measurements on $SrTiO_3(001)$ surfaces having a known lattice constant. Note that these calibrations absorb the overall scaling factor that depends on the working distance between the LEED screen and the sample (and the camera image magnification factor), as well as some higher-order distortions of the spot patterns that result from the sample not being positioned precisely at the center of curvature of the LEED screen (and the screen itself being slightly aspherical).

From these values of $\sin(\theta)$, the electron energies E at which each LEED pattern was recorded, and the (H, K) indices, we compiled lists of lattice constants corresponding to each fitted spot position. For simplicity in analysis, we restricted our attention to spots having $\mathbf{q}_{||}$ purely aligned with [001] or [1$\bar{1}$0]. Elastic scattering and conservation of momentum modulo translations of the surface reciprocal lattice together require that:

$$|\mathbf{q}_{||}| = k\sin(\theta) = \sqrt{2m_e E}\sin(\theta)/\hbar \ , \qquad (8)$$

which for Bragg spots of the type $\mathbf{q}_{||} = 2\pi(H/c, 0)$ and $2\pi(0, K/2d_{1\bar{1}0})$, reduces to:

$$c = \frac{2\pi\hbar H}{\sqrt{2m_e E}\sin(\theta)} \quad \text{and} \quad 2d_{1\bar{1}0} = \frac{2\pi\hbar K}{\sqrt{2m_e E}\sin(\theta)} \ . \qquad (9)$$

Here c and $2d_{1\bar{1}0}$ are the surface lattice constants along [001] and [1$\bar{1}$0] that are expected for an unreconstructed (110)-oriented rutile surface. Histograms of the values obtained in this way for c are displayed in Supplementary Fig. 14a, and the results for $2d_{1\bar{1}0}$ are displayed in Supplementary Fig. 14b. For reference, we also indicate by gray arrows the surface lattice constants expected for bulk-terminated TiO_2 ($c = 2.96$ Å, $2d_{1\bar{1}0} = 6.50$ Å) and bulk-terminated RuO_2 ($c = 3.11$ Å, $2d_{1\bar{1}0} = 6.35$ Å). The surface lattice constants for the 7 nm thick $RuO_2(110)$ sample in blue, ($c = 2.96 \pm 0.03$ Å, $2d_{1\bar{1}0} = 6.51 \pm 0.05$ Å),

match those of TiO_2 within the $\approx 1.0\%$ resolution of the measurements, indicating that this film is (nearly) commensurately strained to the substrate along both [001] and [$1\bar{1}0$]. By contrast, the 48 nm thick $RuO_2(110)$ sample in red shows broader LEED spots, indicating lower surface crystallinity than the 7 nm thick sample, which results in wider distributions of the extracted lattice constants. Furthermore, the centers of mass of the red distributions, ($c = 3.07 \pm 0.06$ Å$, 2d_{1\bar{1}0} = 6.39 \pm 0.11$ Å), are displaced away from the blue distributions towards the values expected for bulk RuO_2; this is why in Fig. 4b-c of the main text we suggest that the surface electronic structure of this 48 nm thick sample measured by ARPES—which probes the film over a comparable depth to LEED, within < 1 nm from the top film surface—should be more representative of bulk RuO_2.

Supplementary Note 8: EXTRACTING THE NEAR-E_F DENSITY OF STATES FROM ARPES MEASUREMENTS

In Supplementary Fig. 15 we present LEED and ARPES data taken on three different RuO_2 thin-film samples: 19 nm thick $RuO_2(101)$, 7 nm thick $RuO_2(110)$, and 48 nm thick $RuO_2(110)$. In Supplementary Fig. 15a, all of the samples show LEED spot patterns with the periodicities expected for unreconstructed (101)- and (110)-oriented rutile surfaces, respectively; furthermore, the sharpness of the patterns suggest high degrees of surface crystallinity, such that in-plane momentum should be a nearly conserved parameter in photoemission. Given that strained $RuO_2(110)$ samples superconduct at measurable T_cs, while $RuO_2(101)$ samples and bulk RuO_2 do not, the question we wanted to address using ARPES was: how does the density of states near the Fermi level, $N(E_F)$, evolve between these samples? Recall that based on the LEED lattice constant analysis described in Supplementary Fig. 14, most of the substrate-imposed epitaxial strains are relaxed at the top surface of the 48 nm thick $RuO_2(110)$ sample, such that its electronic structure probed by ARPES is a reasonable proxy for that of bulk RuO_2. Specifically, the quantity of interest as it relates to the low-energy physics is:

$$N(E_F) \propto \int_{E_F-\delta}^{E_F+\delta} \int_{\mathrm{BZ}} A(\mathbf{k}, \omega) d\mathbf{k} d\omega \ , \tag{10}$$

where $A(\mathbf{k}, \omega)$ is the single-particle spectral function, integrated over all momenta $\mathbf{k}$ in the Brillouin zone (BZ) and over some limited range of energies ω near E_F (δ is some small parameter).

Two separate factors make it extremely challenging to quantitatively extract the total $N(E_F)$ directly from data taken with our lab-based ARPES system. First, our inability to continuously vary the photon energy—or equivalently, the kinetic energy of the photoelectrons at E_F—implies that only regions of the Brillouin zone with specific k_z can be probed, cf. Supplementary Eq. (7). Therefore the full integration over $\mathbf{k}$ in Supplementary Eq. (10) cannot be performed using a lab-based ARPES setup, which is especially problematic in a material such as RuO_2 that has a highly three-dimensional electronic structure depending strongly on k_z (cf. Supplementary Fig. 13). Second, even if the entire Brillouin zone could be mapped exhaustively, the intensity measured in ARPES is not the initial-state spectral function $A(\mathbf{k}, \omega)$, but rather this quantity multiplied by probabilities (i.e., matrix elements) for photoemission, which are difficult to account for theoretically.

With these qualifications in mind, there is a route to answering the simpler question of whether $N(E_F)$ increases in strained $RuO_2(110)$ compared with strained $RuO_2(101)$ or bulk RuO_2: we simply need to determine where the flat bands with $d_{||}$ orbital character are located in energy relative to E_F. DFT calculations suggest that if these bands move closer to (further away from) E_F, the total $N(E_F)$ will increase (decrease), respectively. To approximately determine the positions of these bands experimentally, we integrated the photoemission intensity over the color-coded slabs in the Brillouin zone schematic in Supplementary Fig. 15a, plotted the resulting energy distribution curves (EDCs) in Supplementary Fig. 15b, and found the maxima in the EDCs as indicated by the dashed lines. The regions colored yellow in the Brillouin zone denote where the near-E_F wavefunctions have greater than 90% $d_{||}$ orbital character, according to our DFT + Wannier90 calculations; since all slabs lie in this region, we expect that the dominant contributions to the measured EDCs are from $d_{||}$ initial states. Note that the region of $k_z = k_{110}$ probed by ARPES with He-Iα (21.2 eV) photons on the (110)-oriented samples is well-constrained by analysis of the $E(\mathbf{k})$ dispersions as outlined in Supplementary Fig. 13; however, for the (101)-oriented sample the region of $k_z = k_{101}$ probed by ARPES with He-IIα (40.8 eV) photons is merely calculated from the free-electron final state model in Supplementary Eq. (7), using the same inner potential as for $RuO_2(110)$, and thus is subject to greater experimental uncertainties. Nonetheless, the results of this analysis qualitatively agree with the strain-dependent trends anticipated by DFT (Supplementary Fig. 15c): in highly strained $RuO_2(110)$ films (blue), the flat bands move closer to E_F compared with either more strain-relaxed $RuO_2(110)$ films (red) or commensurately strained $RuO_2(101)$ films (purple). This modification of the effective d orbital degeneracies boosts $N(E_F)$, which—as proposed in the main text—likely contributes to the enhanced superconducting T_cs observed in highly strained $RuO_2(110)$ samples.

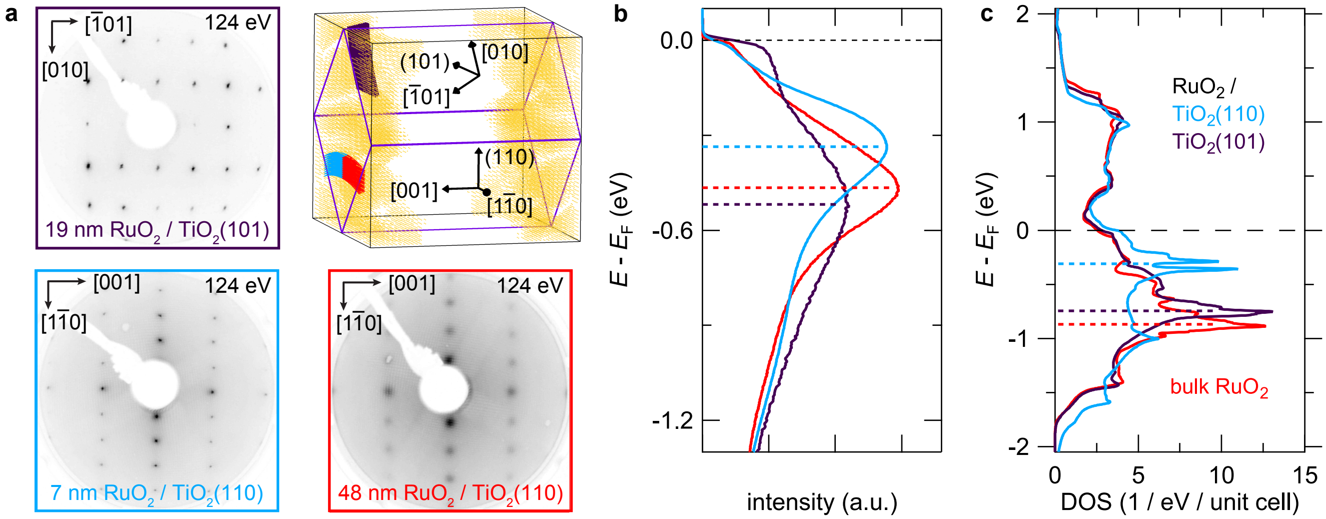

Supplementary Figure 15. **Strain dependence of the near-E_F density of states. a,** LEED patterns recorded at 124 eV for a 19 nm thick $RuO_2(101)$, a 7 nm thick $RuO_2(110)$, and a 48 nm thick $RuO_2(110)$ sample, along with a schematic of the Brillouin zone showing the regions of **k**-space over which we integrated the ARPES data for these samples to obtain the average energy distribution curves shown in **b**. Regions of the Brillouin zone shaded yellow indicate where the near-E_F Kohn-Sham wavefunctions have $>$ 90% $d_{||}$ orbital character. **b,** The colored dashed lines represent the peak positions of the energy distribution curves. By locating the $d_{||}$-derived flat bands in this way, trends in how $N(E_F)$ evolves with strain can be more reliably extracted from ARPES data than by directly reading off the measured photoemission intensity at E_F. **c,** Strain-dependent changes in the total DOS expected according to DFT + U simulations, reproduced from Fig. 4a.

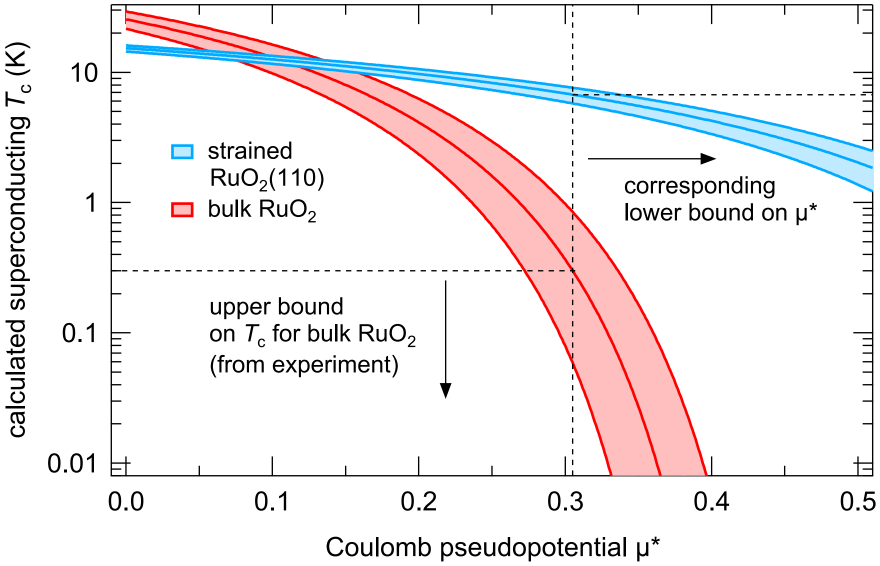

Supplementary Figure 16. **Calculated T_cs for bulk RuO_2 and strained $RuO_2(110)$, assuming an electron-phonon mediated mechanism of superconductivity.** T_c is evaluated from Supplementary Eq. (11) using DFT-based calculations of $\lambda_{\text{el-ph}}$ and $\omega_{\log}$, and allowing μ^* to vary. As indicated by the vertical and horizontal dashed lines, $\mu^* > 0.30$ is the region of parameter space consistent with the reported non-superconducting behavior of bulk RuO_2 ($T_c < 0.3$ K [39]), which in turn places an upper bound ($T_c < 7$ K) on the strain-enhanced T_cs expected for $RuO_2(110)$. Shaded regions indicate the changes in T_c that would result from ±10% errors in the calculated values of $\lambda_{\text{el-ph}}$.

Supplementary Note 9: MIGDAL-ELIASHBERG CALCULATIONS OF T_c

As described in the Methods section of the main text, we performed first-principles DFT-based electron-phonon coupling calculations of the isotropic Eliashberg spectral function $\alpha^2F(\omega)$ and total electron-phonon coupling constant $\lambda_{\text{el-ph}}$ (integrated over all phonon modes and wavevectors) for bulk RuO_2 and commensurately strained $RuO_2(110)$. From these quantities, we estimated the superconducting transition temperatures using the semi-empirical McMillan-Allen-Dynes formula:

$$T_c = \frac{\omega_{\log}}{1.2} \exp\left[-\frac{1.04(1+\lambda_{\text{el-ph}})}{\lambda_{\text{el-ph}} - \mu^*(1+0.62\lambda_{\text{el-ph}})}\right] \qquad (11)$$

McMillan obtained a formula resembling Supplementary Eq. (11) by numerically solving the equations of finite-temperature Migdal-Eliashberg theory using the experimentally measured spectral function of niobium [40]; Allen and Dynes improved the agreement of McMillan's formula with experimentally measured $\alpha^2F(\omega)$ and T_cs for a variety of conventional superconductors by introducing an appropriately weighted average over $\alpha^2F(\omega)$ in the prefactor of the exponential, rather than using the Debye temperature [41]. In essence, Supplementary Eq. (11) can be considered an extension of Eq. (1) from the main text that identifies phonons as the bosonic modes that mediate Cooper pairing (i.e., $\lambda = \lambda_{\text{el-ph}}$), and which remains valid even in the limit of stronger couplings (i.e., $\lambda > 1$) by virtue of Migdal's theorem for the electron-phonon interaction.

For bulk RuO_2 (strained $RuO_2(110)$, respectively), we obtained $\lambda_{\text{el-ph}} = 0.685$ and $\omega_{\log} = 34.1$ meV ($\lambda_{\text{el-ph}} = 1.97$ and $\omega_{\log} = 7.59$ meV). The large strain-induced enhancement in $\lambda_{\text{el-ph}}$ and shift of $\omega_{\log}$ to lower frequencies is caused by substantial phonon softening that occurs under c-axis compression in the rutile structure. In fact, we found that some of the calculated zone-boundary phonon frequencies even become imaginary under strain in $RuO_2(110)$, possibly indicating an incipient structural instability. A more detailed account of this phenomenon will be described in a future publication; for the purposes of this work, we omitted such phonon modes in subsequent electron-phonon coupling calculations, and neglected any errors this may cause in $\lambda_{\text{el-ph}}$ and $\omega_{\log}$.

To convert the calculated values of $\lambda_{\text{el-ph}}$ and $\omega_{\log}$ to T_cs via Supplementary Eq. (11) requires knowledge of the appropriate value(s) of the screened Coulomb interaction μ^* between quasiparticles. Typically μ^* is chosen in an ad hoc fashion to match the experimentally measured T_c of a given material. Because bulk RuO_2 is not known to be superconducting at experimentally accessible temperatures, we cannot employ such a prescription here; nevertheless, we can use the experimentally measured least upper bound on T_c for bulk RuO_2 ($T_c < 0.3$ K [39]) to place a lower bound on μ^* ($\mu^* > 0.30$), as illustrated by the dashed lines in Supplementary Fig. 16. For this range of μ^*, inserting the values of $\lambda_{\text{el-ph}}$ and $\omega_{\log}$ calculated for $RuO_2(110)$ into Supplementary Eq. (11) predicts $T_c < 7$ K, which agrees reasonably well with the experimentally measured values. Because several uncontrolled approximations enter into these estimates of T_c, we consider this level of agreement as suggestive, although not conclusive, evidence for a phonon-mediated mechanism of superconductivity; in any case, it is clear that reducing the axial ratio c/a in appropriately strained variants of RuO_2 robustly boosts $\lambda_{\text{el-ph}}$, in good agreement with expectations based on steric trends for other rutile compounds [42, 43]. Any effects of strain on μ^* are ignored for the purposes of this work.

[1] Miccoli, I., Edler, F., Pfnuer, H. & Tegenkamp, C. The 100th anniversary of the four-point probe technique: the role of probe geometries in isotropic and anisotropic systems. *Journal of Physics: Condensed Matter* **27**, 223201 (2015).

[2] Ryden, W. D., Lawson, A. W. & Sartain, C. C. Electrical Transport Properties of IrO_2 and RuO_2. *Physical Review B* **1**, 1494–1500 (1970).

[3] Glassford, K. M. & Chelikowsky, J. R. Electron transport properties in RuO_2 rutile. *Physical Review B* **49**, 7107–7114 (1994).

[4] Wolff, K. *et al.* Influence of the vicinal substrate miscut on the anisotropic two-dimensional electronic transport in Al_2O_3–$SrTiO_3$ heterostructures. *Journal of Applied Physics* **128**, 085302 (2020).

[5] Wu, J. *et al.* Electronic nematicity in Sr_2RuO_4. *Proceedings of the National Academy of Sciences* **117**, 10654–10659 (2020).

[6] Schneider, C. W., Thiel, S., Hammerl, G., Richter, C. & Mannhart, J. Microlithography of electron gases formed at interfaces in oxide heterostructures. *Applied Physics Letters* **89**, 122101 (2006).

[7] Hsu, J. W. P. & Kapitulnik, A. Superconducting transition, fluctuation, and vortex motion in a two-dimensional single-crystal Nb film. *Physical Review B* **45**, 4819–4835 (1992).

[8] Kim, M., Kozuka, Y., Bell, C., Hikita, Y. & Hwang, H. Y. Intrinsic spin-orbit coupling in superconducting δ-doped $SrTiO_3$ heterostructures. *Physical Review B* **86**, 085121 (2012).

[9] Werthamer, N. R., Helfand, E. & Hohenberg, P. C. Temperature and Purity Dependence of the Superconducting Critical Field, H_{c2}. III. Electron Spin and Spin-Orbit Effects. *Physical Review* **147**, 295–302 (1966).

[10] Wissberg, S. & Kalisky, B. Large-scale modulation in the superconducting properties of thin films due to domains in the $SrTiO_3$ substrate. *Physical Review B* **95**, 144510 (2017).

[11] Kalisky, B. *et al.* Stripes of increased diamagnetic susceptibility in underdoped superconducting $Ba(Fe_{1-x}Co_x)_2As_2$ single crystals: Evidence for an enhanced superfluid density at twin boundaries. *Physical Review B* **81**, 184513 (2010).

[12] Kalisky, B. *et al.* Behavior of vortices near twin boundaries in underdoped $Ba(Fe_{1-x}Co_x)_2As_2$. *Physical Review B* **83**, 064511 (2011).

[13] Berlijn, T. *et al.* Itinerant Antiferromagnetism in RuO_2. *Physical Review Letters* **118**, 077201 (2017).

[14] Burdett, J. K., Hughbanks, T., Miller, G. J., Richardson, J. W. & Smith, J. V. Structural-electronic relationships in inorganic solids: powder neutron diffraction studies of the rutile and anatase polymorphs of titanium dioxide at 15 and 295 K. *Journal of the American Chemical Society* **109**, 3639–3646 (1987).

[15] Miceli, P. F. & Palmstrom, C. J. X-ray scattering from rotational disorder in epitaxial films: An unconventional mosaic crystal. *Physical Review B* **51**, 5506–5509 (1995).

[16] Miceli, P. F., Weatherwax, J., Krentsel, T. & Palmstrom, C. J. Specular and diffuse reflectivity from thin films containing misfit dislocations. *Physica B: Condensed Matter* **221**, 230–234 (1996).

[17] Kortan, A. R., Hong, M., Kwo, J., Mannaerts, J. P. & Kopylov, N. Structure of epitaxial Gd_2O_3 films grown on GaAs(100). *Physical Review B* **60**, 10913–10918 (1999).

[18] Barabash, R. I., Donner, W. & Dosch, H. X-ray scattering from misfit dislocations in heteroepitaxial films: The case of Nb(110) on Al_2O_3. *Applied Physics Letters* **78**, 443–445 (2001).

[19] Biegalski, M. D. *et al.* Critical thickness of high structural quality $SrTiO_3$ films grown on orthorhombic (101) $DyScO_3$. *Journal of Applied Physics* **104**, 114109 (2008).

[20] Wang, T., Ganguly, K., Marshall, P., Xu, P. & Jalan, B. Critical thickness and strain relaxation in molecular beam epitaxy-grown $SrTiO_3$ films. *Applied Physics Letters* **103**, 212904 (2013).

[21] Kaganer, V. M., Koehler, R., Schmidbauer, M., Opitz, R. & Jenichen, B. X-ray diffraction peaks due to misfit dislocations in heteroepitaxial structures. *Physical Review B* **55**, 1793–1810 (1997).

[22] Csiszar, S. I. & Tjeng, L. H. *X-ray diffraction and X-ray absorption of strained CoO and MnO thin films.* Ph.D. thesis, University of Groningen (2005).

[23] Xie, S. *et al.* Coherent, atomically thin transition-metal dichalcogenide superlattices with engineered strain. *Science* **359**, 1131–1136 (2018).

[24] Bachmann, M. D. *et al.* Spatial control of heavy-fermion superconductivity in $CeIrIn_5$. *Science* **366**, 221–226 (2019).

[25] Warusawithana, M. P. *et al.* A Ferroelectric Oxide Made Directly on Silicon. *Science* **324**, 367–370 (2009).

[26] Glatz, A., Aranson, I. S., Baturina, T. I., Chtchelkatchev, N. M. & Vinokur, V. M. Self-organized superconducting textures in thin films. *Physical Review B* **84**, 024508 (2011).

[27] Eley, S., Gopalakrishnan, S., Goldbart, P. M. & Mason, N. Approaching zero-temperature metallic states in mesoscopic superconductor–normal–superconductor arrays. *Nature Physics* **8**, 59–62 (2012).

[28] Pinto, N. *et al.* Dimensional crossover and incipient quantum size effects in superconducting niobium nanofilms. *Scientific Reports* **8**, 4710 (2018).

[29] Meyer, T. L., Jiang, L., Park, S., Egami, T. & Lee, H. N. Strain-relaxation and critical thickness of epitaxial $La_{1.85}Sr_{0.15}CuO_4$ films. *APL Materials* **3**, 126102 (2015).

[30] Kapitulnik, A., Kivelson, S. A. & Spivak, B. Colloquium: Anomalous metals: Failed superconductors. *Reviews of Modern Physics* **91**, 011002 (2019).

[31] Haviland, D. B., Liu, Y. & Goldman, A. M. Onset of superconductivity in the two-dimensional limit. *Physical Review Letters* **62**, 2180–2183 (1989).

[32] Jaeger, H. M., Haviland, D. B., Orr, B. G. & Goldman, A. M. Onset of superconductivity in ultrathin granular metal films. *Physical Review B* **40**, 182–196 (1989).

[33] Steiner, M. A., Breznay, N. P. & Kapitulnik, A. Approach to a superconductor-to-Bose-insulator transition in disordered films. *Physical Review B* **77**, 212501 (2008).

[34] Mackenzie, A. P. *et al.* Extremely Strong Dependence of Superconductivity on Disorder in Sr_2RuO_4. *Physical Review Letters* **80**, 161–164 (1998).

[35] Kresse, G. & Furthmueller, J. Efficient iterative schemes for ab initio total-energy calculations using a plane-wave basis set. *Physical Review B* **54**, 11169–11186 (1996).

[36] Kresse, G. & Joubert, D. From ultrasoft pseudopotentials to the projector augmented-wave method. *Physical Review B* **59**, 1758–1775 (1999).

[37] Zhu, Z. *et al.* Anomalous Antiferromagnetism in Metallic RuO_2 Determined by Resonant X-ray Scattering. *Physical Review Letters* **122**, 017202 (2019).

[38] Ahn, K.-H., Hariki, A., Lee, K.-W. & Kunes, J. Antiferromagnetism in RuO_2 as *d*-wave Pomeranchuk instability. *Physical Review B* **99**, 184432 (2019).

[39] Lin, J. J. *et al.* Low temperature electrical transport properties of RuO_2 and IrO_2 single crystals. *Journal of Physics: Condensed Matter* **16**, 8035 (2004).

[40] McMillan, W. L. Transition Temperature of Strong-Coupled Superconductors. *Physical Review* **167**, 331–344 (1968).

[41] Allen, P. B. & Dynes, R. C. Transition temperature of strong-coupled superconductors reanalyzed. *Physical Review B* **12**, 905–922 (1975).

[42] Eyert, V. The metal-insulator transitions of VO_2: A band theoretical approach. *Annalen der Physik* **11**, 650–704 (2002).

[43] Hiroi, Z. Structural instability of the rutile compounds and its relevance to the metal–insulator transition of VO_2. *Progress in Solid State Chemistry* **43**, 47–69 (2015).
