# Supplementary material for: Strain-stabilized superconductivity
Source: Nat Commun. 2021 Jan 4;12:59. doi: 10.1038/s41467-020-20252-7 (PMC7782483; doi:10.1038/s41467-020-20252-7)
Supplement: Supplementary file 2 — Peer Review File [file 41467_2020_20252_MOESM2_ESM.docx]

**Editorial Note:** This manuscript has been previously reviewed at another journal that is not operating a transparent peer review scheme. The manuscript was considered suitable for publication without further review at *Nature Communications.*
